# An extensible big data software architecture managing a research resource of real-world clinical radiology data linked to other health data from the whole Scottish population

**DOI:** 10.1093/gigascience/giaa095

**Published:** 2020-09-29

**Authors:** Thomas Nind, James Sutherland, Gordon McAllister, Douglas Hardy, Ally Hume, Ruairidh MacLeod, Jacqueline Caldwell, Susan Krueger, Leandro Tramma, Ross Teviotdale, Mohammed Abdelatif, Kenny Gillen, Joe Ward, Donald Scobbie, Ian Baillie, Andrew Brooks, Bianca Prodan, William Kerr, Dominic Sloan-Murphy, Juan F R Herrera, Dan McManus, Carole Morris, Carol Sinclair, Rob Baxter, Mark Parsons, Andrew Morris, Emily Jefferson

**Affiliations:** Health Informatics Centre (HIC), School of Medicine, University of Dundee, (Main level 5 corridor), Second Floor, Level 7, Mailbox 15, Ninewells Hospital & Medical School, Dundee DD1 9SY2, UK; Health Informatics Centre (HIC), School of Medicine, University of Dundee, (Main level 5 corridor), Second Floor, Level 7, Mailbox 15, Ninewells Hospital & Medical School, Dundee DD1 9SY2, UK; Health Informatics Centre (HIC), School of Medicine, University of Dundee, (Main level 5 corridor), Second Floor, Level 7, Mailbox 15, Ninewells Hospital & Medical School, Dundee DD1 9SY2, UK; Health Informatics Centre (HIC), School of Medicine, University of Dundee, (Main level 5 corridor), Second Floor, Level 7, Mailbox 15, Ninewells Hospital & Medical School, Dundee DD1 9SY2, UK; Edinburgh Parallel Computing Centre (EPCC), Edinburgh University, Bayes Centre, 47 Potterrow, Edinburgh EH8 9BT, UK; Edinburgh Parallel Computing Centre (EPCC), Edinburgh University, Bayes Centre, 47 Potterrow, Edinburgh EH8 9BT, UK; Electronic Data Research and Innovation Service (eDRIS), Public Health Scotland (PHS), Nine, Edinburgh Bioquarter, Little France Road, Edinburgh EH16 4UX, UK; Health Informatics Centre (HIC), School of Medicine, University of Dundee, (Main level 5 corridor), Second Floor, Level 7, Mailbox 15, Ninewells Hospital & Medical School, Dundee DD1 9SY2, UK; Health Informatics Centre (HIC), School of Medicine, University of Dundee, (Main level 5 corridor), Second Floor, Level 7, Mailbox 15, Ninewells Hospital & Medical School, Dundee DD1 9SY2, UK; Health Informatics Centre (HIC), School of Medicine, University of Dundee, (Main level 5 corridor), Second Floor, Level 7, Mailbox 15, Ninewells Hospital & Medical School, Dundee DD1 9SY2, UK; Health Informatics Centre (HIC), School of Medicine, University of Dundee, (Main level 5 corridor), Second Floor, Level 7, Mailbox 15, Ninewells Hospital & Medical School, Dundee DD1 9SY2, UK; Health Informatics Centre (HIC), School of Medicine, University of Dundee, (Main level 5 corridor), Second Floor, Level 7, Mailbox 15, Ninewells Hospital & Medical School, Dundee DD1 9SY2, UK; Health Informatics Centre (HIC), School of Medicine, University of Dundee, (Main level 5 corridor), Second Floor, Level 7, Mailbox 15, Ninewells Hospital & Medical School, Dundee DD1 9SY2, UK; Edinburgh Parallel Computing Centre (EPCC), Edinburgh University, Bayes Centre, 47 Potterrow, Edinburgh EH8 9BT, UK; Electronic Data Research and Innovation Service (eDRIS), Public Health Scotland (PHS), Nine, Edinburgh Bioquarter, Little France Road, Edinburgh EH16 4UX, UK; Edinburgh Parallel Computing Centre (EPCC), Edinburgh University, Bayes Centre, 47 Potterrow, Edinburgh EH8 9BT, UK; Edinburgh Parallel Computing Centre (EPCC), Edinburgh University, Bayes Centre, 47 Potterrow, Edinburgh EH8 9BT, UK; Edinburgh Parallel Computing Centre (EPCC), Edinburgh University, Bayes Centre, 47 Potterrow, Edinburgh EH8 9BT, UK; Edinburgh Parallel Computing Centre (EPCC), Edinburgh University, Bayes Centre, 47 Potterrow, Edinburgh EH8 9BT, UK; Edinburgh Parallel Computing Centre (EPCC), Edinburgh University, Bayes Centre, 47 Potterrow, Edinburgh EH8 9BT, UK; Edinburgh Parallel Computing Centre (EPCC), Edinburgh University, Bayes Centre, 47 Potterrow, Edinburgh EH8 9BT, UK; Electronic Data Research and Innovation Service (eDRIS), Public Health Scotland (PHS), Nine, Edinburgh Bioquarter, Little France Road, Edinburgh EH16 4UX, UK; Data Driven Innovation, Public Health Scotland (PHS), Gyle Square, 1 South Gyle Crescent, Edinburgh EH12 9EB, UK; Edinburgh Parallel Computing Centre (EPCC), Edinburgh University, Bayes Centre, 47 Potterrow, Edinburgh EH8 9BT, UK; Edinburgh Parallel Computing Centre (EPCC), Edinburgh University, Bayes Centre, 47 Potterrow, Edinburgh EH8 9BT, UK; Health Data Research (HDR) UK, Gibbs Building, 215 Euston Road, London NW1 2BE, UK; Health Informatics Centre (HIC), School of Medicine, University of Dundee, (Main level 5 corridor), Second Floor, Level 7, Mailbox 15, Ninewells Hospital & Medical School, Dundee DD1 9SY2, UK

**Keywords:** Radiology, Big Data, AI, ML

## Abstract

**Aim:**

To enable a world-leading research dataset of routinely collected clinical images linked to other routinely collected data from the whole Scottish national population. This includes more than 30 million different radiological examinations from a population of 5.4 million and >2 PB of data collected since 2010.

**Methods:**

Scotland has a central archive of radiological data used to directly provide clinical care to patients. We have developed an architecture and platform to securely extract a copy of those data, link it to other clinical or social datasets, remove personal data to protect privacy, and make the resulting data available to researchers in a controlled Safe Haven environment.

**Results:**

An extensive software platform has been developed to host, extract, and link data from cohorts to answer research questions. The platform has been tested on 5 different test cases and is currently being further enhanced to support 3 exemplar research projects.

**Conclusions:**

The data available are from a range of radiological modalities and scanner types and were collected under different environmental conditions. These real-world, heterogenous data are valuable for training algorithms to support clinical decision making, especially for deep learning where large data volumes are required. The resource is now available for international research access. The platform and data can support new health research using artificial intelligence and machine learning technologies, as well as enabling discovery science.

## Background

### Advantages and challenges of using routinely collected clinical images for research

Clinical images, especially when linked to other routinely collected health data, are extremely useful for many types of research: examining early/preclinical diagnosis [1], disease progression [2, 3], genotype-phenotype associations [4], development of risk profiles [5, 6], computer vision methods for biomarker extraction [7–9], machine learning approaches [10–13], and discovery and classification of disease types [14]. The emerging field of radiomics has the potential to bridge the gap between medical imaging and personalised medicine [15]. However, collecting images for specific research projects is expensive and constrains the scale of many studies. Research cohorts are usually composed of a narrow subset of people with a specific condition, which can make both generalising findings and repurposing of images for research problematic. Use of routinely collected images, in contrast, opens up the potential for very large-scale studies, which not only efficiently and effectively complement smaller disease-based cohorts of patients but are also extremely flexible when linked to extensive electronic medical records, allowing for a wide range of disease areas to be examined. However, whereas research images are typically collected using specific image acquisition protocols under ideal conditions, routinely collected clinical images are more heterogeneous.

Using clinical images for research and linking them to other routinely collected clinical data is challenging because:

1. Existing software used to query/search for images from the Picture Archive Communication System (PACS) is designed for clinical care rather than research. The software makes it easy to find all images for a particular patient but is not designed to facilitate searching for all images with particular characteristics such as body part; slice thickness, scanning protocol, contrast agent, or patient medication; or linking to other Electronic Health Record (EHR) datasets (e.g., outcome data, prescription data).
2. Re-use of clinical images for research required de-identification. However, identifiable data can be present in many areas of the associated DICOM (Digital Imaging and Communications in Medicine, RRID:SCR_018878) [16] file metadata and/or may be present within the pixel data themselves, “burned on” to the actual image.
3. Anonymisation of images can reduce the ability to perform linkage to other datasets, e.g., demography, prescribing, hospital admissions.
4. Reuse often requires approval from multiple data controllers, and the complexity of de-identification increases the risk of rejection of applications for research given the amount of work the data controller may have to do to ensure that no identifiable data are released.
5. For deep learning projects, where large numbers of images are required, the image extraction costs for research can be prohibitive.

### Scottish clinical and research data

#### Scottish clinical PACS system

Scotland has a single National PACS Clinical System that contains all radiological images collected from 14 different health boards. To date (2019), this includes 30 million different radiological examinations from a population of 5.6 million and >2 PB of data collected since 2010. It includes a range of modalities (including computed tomography [CT], MRI, ultrasound, nuclear medicine imaging, and plain film radiography). This system is a live environment used directly for clinical care.

#### Provision of routinely collected text-based clinical data for research

Scotland has a relatively stable population with long-established use of a unique healthcare identifier (the Community Health Index [CHI] number) that is also increasingly seeded in data in other sectors such as social care. A National Health Service (NHS) Scotland service, called the electronic Data Research and Innovation Service (eDRIS) [17], provides a National Safe Haven environment (hosted by the University of Edinburgh) to support research access to anonymised extracts of linked data from different data controllers to answer specific approved research questions. The linkable phenotypic data include a range of national datasets including, e.g., prescribing, death data, and hospital admissions.

Subject to robust pseudonymisation safeguards and approval by the Public Benefit and Privacy Panel for Health and Social Care, individual patient consent is not required in Scotland. This project assembles a library of imaging data and then generates thoroughly redacted subsets for research projects. Multiple safeguards are applied. The subsets are themselves only released to approved research projects within the controlled environment of the National Safe Haven computers; any extraction of data beyond that is subject to further controls to protect the privacy of patients.

The technical safeguards described in this article and implemented in this project are not perfect and are not the sole protection: rare medical conditions or identifying features may still be present within the research extracts generated. Contractual and administrative precautions manage these risks: researchers are both contractually prohibited from attempting to re-identify patients or link against unapproved datasets and prevented from exporting the raw data beyond the confines of the Safe Haven because any such export could enable such an attempt to be made.

#### Incorporating clinical imaging data into the wealth of available datasets for research

A research copy of the data held within the Scottish National Clinical PACS system has been created to enable the clinical imaging data to be linked with the other routinely collected datasets and be made accessible for research (given appropriate data governance approvals). The research copy of the Clinical PACS system is called the Scottish Medical Imaging (SMI) Database. The data are held in the non-proprietary DICOM format.

The management of imaging data for research presents a substantial set of challenges beyond those encountered in the management of purely text-based records. Some of these are variations on familiar challenges, such as de-identification, whilst others are novel and intrinsic to this type of dataset, such as size and compute requirements for big data processing.

This article describes the architectural solution and software platform developed to support hosting, extracting, and linking the SMI data, which addresses the aforementioned challenges identified of using routinely collected imaging data for research.

We first describe the project approach, a very high level summary of the requirements, then our architectural solution, an explanation of why this solution met the requirements and why our solution is different to that of other open-source solutions for the large-scale hosting of imaging data. We explain how the architecture enables feedback and enhancement improvements from other sources. We then describe our progress towards implementing the architecture and the use cases we have tested.

## Project Approach

There have been 4 phases to the project to date.

### Requirement gathering

An initial requirement gathering exercise was undertaken at the project inception eliciting requirements from the research community who will use the data extracts provided by the platform, the National Health Service Data Governance representatives as the data controllers of the data, and the National Safe Haven staff who will use the platform to build cohorts and provision relevant data extracts to researchers for analysis. We also investigated other open source and freely available platforms for hosting and/or anonymising imaging data to see if any of these could be used entirely or in part within our solution. We researched both functional and non-functional requirements of the solution.

### Development of the architecture

We developed a range of option appraisals and designed an architectural solution to meet the requirements.

### Development of prototype

We developed prototype software to run in a Regional Safe Haven environment managed by the University of Dundee, whilst the SMI data transfer project was taking place in parallel. This prototype supported 2 consented research projects, predicting dementia from CT and MRI images. We then expanded the prototype to run in the National Safe Haven.

### Testing and case studies on sample data

The software was then tested on a 180-TB subset of the full dataset including ∼3 million studies (which were loaded into the Document Store—see below). These were images generated across Scotland during the same 2-week period in February for each year in a 7-year period. Linkage, extraction, and anonymisation was performed for a range of case studies along with performance and functional testing. (Case studies are listed in Online Appendix D and included linking against the Scottish Cancer Registry's SMR06 diagnosis data and Radiology Information System records, as well as the DICOM metadata.) The full set of historical data was still in the process of being decrypted from the proprietary PACS vendor format and could therefore not be used for complete testing at this stage.

## Platform Architecture

A list of high-level platform requirements is provided in Online Appendix A.

### Architecture overview

**Figure 1:**
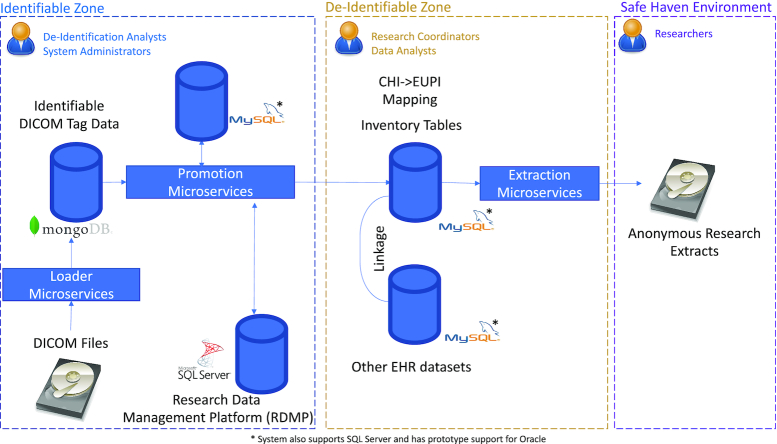
Overview of the architecture. There are 3 zones: the identifiable zone (which holds the raw data), the de-identified zone (where cohort building and linking to other data takes place) and the Safe Haven environment (where a researcher carries out their analysis).

The high-level platform architecture is shown in Fig. 1. The Discussion section describes our future plans.

The SMI Data Repository is divided into identifiable and de-identified zones. The SMI Analytic Platform is the Safe Haven environment where researchers can access their relevant data extracts.

Each zone has audited, controlled access with a clear separation of roles and functions. Only system administrators can access the identifiable zone to carry out maintenance and security functions. Only de-identification analysts can view the potentially identifiable data in their duties. Research coordinators can query de-identified text-based (including DICOM tag values) data for cohort building, linkage, and extraction in the de-identified zone. Researchers can access de-identified (using Project-IDs) metadata and de-identified pixel data for their cohort within the Virtual Machine (VM) Safe Haven environment.

The CHI number encodes potentially identifiable information such as date of birth, so we use an encrypted version known as EUPI (Encrypted Universal Patient Identifier) within the de-identified zone, and this is typically used for patient-linking tasks.

Two processes (historic and ongoing) extract DICOM files from the proprietary PACS format received from the NHS. These DICOM files contain identifiable metadata (DICOM Tags) that are read and stored in full in the Document Store (a MongoDB database) by the Loader. The DICOM files also contain the pixel image information.

The "Identifiable DICOM Tag Data" are analysed for potentially identifiable data. Relative file paths and a subset of DICOM Metadata that can be de-identified and is useful for cohort building are sent to the "inventory tables" (a relational database) and stored using EUPIs within the Identifiable Zone. A mirror of the Inventory Tables is provided to data analysts in the de-identified zone. This mirror supports condensing tag data, e.g., to study/series level to improve performance of cohort generation/linkage.

The inventory tables can be queried like any other database by research co-coordinators to construct cohorts using their existing working procedures. Once a research extract has been created, the corresponding image UIDs (study/series or SOPInstanceUID) are supplied to the extraction process. The extraction process uses the inventory tables to locate and anonymise the DICOM files held in the data store. These anonymised files are then provided to the researcher in the Safe Haven environment.

A summary of the expected functionality of the data stores and process within the architecture is provided in Tables 1 and 2.

**Table 1:** Overview of data stores

Data	Description
DICOM files (unmodified historic data)	DICOM files are stored unaltered in a file archive. There are many reasons why we wish to keep the identifiable data and store the original DICOM images:• Any program developed to strip all identifiable data from the DICOM files and tags risks rendering the whole dataset unusable if this is done incorrectly. Linkage to other datasets would subsequently be either incorrect or impossible.• It is conceivable that future data de-identification strategies will wish to make use of some identifiable data and removing that data would therefore limit future options.• The NHS may wish to use the data as a secondary offline disaster recovery system or use the data to populate a clinical system from an alternative provider. In this case it needs to be technically feasible to generate the data in identifiable form in a format that is non-proprietary and as close as possible to the DICOM files as they were originally captured.
Identifiable DICOM tag data	All tag metadata from the DICOM files are extracted to a MongoDB database in a searchable format. These tag metadata are stored in an identifiable format because de-identification analysts need to know what the identifiable data are so that they can remove them, e.g.,• If the patient name is Mrs Jones, then a de-identification analyst searching for identifiable data in the clinical report will need to know to look for the text “Jones” in order to remove it.• To check whether an image is identifiable, the de-identification analyst might need to know the CHI number in order to check that it is not burnt into the pixel data.
Inventory tables	A subset of data from the Identifiable set above is copied here. This is a relational database that contains suitably cleansed and de-identified image metadata (and file paths), i.e., has been confirmed to be well populated, of high quality, and does not contain identifiable data. This is used by the research co-ordinators for cohort creation and extraction to the Safe Haven. The data are indexed using EUPIs.For example, DICOM age strings can express the age in years, months, or days (e.g., 075Y, 006M, or 002D). The cleaned and homogenized metadata will store these in a consistent and easily queried numeric format. Other metadata fields may be a single value summarising data stored in multiple different DICOM tags. For example, by analysing the acquisition position of the images it is possible to identify examinations in which the same volume has been acquired repeatedly in a single series; when used in conjunction with tags that indicate whether contrast was used during the examination, this can be used to disambiguate contrast bolus imaging from other acquisitions that may also use contrast.
Cohort and associated anonymous research extracts	Any research project will start by defining a relevant cohort and obtaining the necessary ethical/administrative approval in consultation between the researchers and the research coordinators—this is an out-of-band process outwith the iRDMP system, so not shown here. The data analysts then assemble a dataset (the anonymous research extract) for that research project by querying the inventory table, possibly linked against other data sources via the EUPI (pseudonymised patient ID, explained below) and trigger the Extraction Microservices to export the appropriate subset of columns made available to the research users. For example, a project might request all available brain MRI scans from patients who have been prescribed gabapentin and want the dosage information and patient age; they would be given a set of image data (the scans themselves, passed through the DICOM file anonymiser described later) and a table of associated metadata including the dosage information for each de-identified patient.
Research Data Management Platform (RDMP)	The RDMP manages and monitors the extraction processes.
CHI to EUPI mapping table	Scotland uses the CHI unique identifier for health data. Adhering to the guiding principles of data linkage for research [14], the National Safe Haven separates out the roles of indexer and linker. Research co-ordinators link data from a range of sources provided to them with the CHI replaced by the EUPI identifier. The imaging data also follow this methodology. The mapping table is securely held and is only accessible via the automated conversion process. An individual can be given multiple CHIs if they access healthcare in different regions, and it takes time to reconcile; therefore the mapping table is updated monthly.

**Table 2:** Overview of Processes

Processes	Description
Promotion of de-identifiable tags/metadata	Promotion is a 2-stage process. The first stage promotes images for which there is an anonymisation protocol (e.g., CT images). Anonymised tag data are pushed to the inventory table. These tables support the extraction processes and support routine practices, e.g., data cleaning. A subset of these data (e.g., only primary/original images) is pushed to the de-identified zone, indicating that the images can be used for cohort generation/image extraction. This data push can include collapsing data, e.g., to series/study level. It is not feasible or desirable to proactively analyse the complete identifiable DICOM tag data in order to promote all tags. This is in part due to the difficulty in determining that a tag of a certain type does contain identifiable information for (i) the whole of the current archive and (ii) future PACS images that will be taken. A tag can be promoted in 2 circumstances: (1) it is determined not to contain identifiable information or (2) the identifiable information it does contain can be de-identified. Sophisticated techniques such as natural language processing (NLP) methodologies can be used to determine Condition 1 or find a solution for de-identification for Condition 2. The solution for Condition 2 is known as an anonymisation profile and can be saved for reuse. Once a tag can be flagged as safe for promotion it is moved to the inventory table. This is an iterative process (future studies with unique requirements will inform which data are prioritised for anonymisation/promoted).
Promotion of image types that are extractable	This process whitelists images that are extractable in the sense that pixel data can completely be de-identified. Some images, particularly ultrasounds, may have identifiable information such as patient name or CHI watermarked on the image. Which images can be de-identified is stored in the metadata catalogue, but the rules regarding how images are de-identified are stored in CTP [17] anonymisation scripts that apply to all images. These scripts contain rules such as “if Modality is US and Manufacturer is X and model is Y then blank out pixels in the rectangle (0 0 1000 200).”
Mapping (CHI-EUPI)	This process is called when metadata are promoted to the de-identifiable zone to replace identifiable CHIs with EUPI. It is an automated process so that no individuals can see this mapping.
Cohort creation process	A set of software tools (or manual SQL queries if the user prefers) that query the DICOM metadata within the inventory tables to select images relevant for a particular cohort (by applying filters that describe researcher requirements). The resulting cohort forms the basis for both the initial and subsequent releases of data to the Safe Haven for the relevant study, and as such it is critically important that the cohort be identified and managed correctly.
Extraction process	This process uses the cohort database and inventory tables to determine which files to extract for a particular research project. It calls the DICOM file anonymiser to de-identify the relevant files used to build the cohort for release to the researcher. After the cohort output and the de-identified DICOM files are curated, the process triggers a release into the researcher Safe Haven environment.
DICOM file anonymiser	
	The DICOM file anonymiser:
	• Obtains the file(s) from the file archives
	• Anonymises the pixel data of the file if necessary
	• Anonymises the metadata in the file (leaving only the whitelisted tags)
	• Converts the file to an alternative format if required
	• Returns the final file(s) to the user
Researcher VM with tools to view and manipulate images	There are 2 main use cases: small-scale studies in which a researcher team may wish to open and mark up each image by eye and large-scale studies in which software and algorithms will be developed by the users of the system to analyse the images for their specific project. The different tools available within the Safe Haven meet both sets of requirements. The researcher VM image includes a standard set of tools, which will be increased over time as the requirements increase. Example tools are MicroDICOM (simple DICOM viewer), ClearCanvas (open-source PACS client, cf. Carestream), and XNAT. The VM should have the capability for users to securely add their own tools. The VM provides access to the associated data from study-specific image metadata and pixel data but does not allow row-level or pixel data to be extracted. Access to the internet is restricted when analysing the data.

**Table 3:** Roles

Role	Description
Researchers	Carry out the research on a dataset extracted from the SMI DB and other linked data. Any project may have a variety of researchers including clinicians, statisticians, radiographers, image analysis and machine learning experts, and so forth. They view and work on the PACS images within a Safe Haven environment.
Research coordinators/cohort builders	Work with the researchers to produce the data extract that allows the research study to be carried out. Research coordinators understand where the data are stored and how to link across datasets and will run software, write scripts, query databases, etc., to produce the final cohort datasets.
Data analysts	Work with de-identified PACS data to produce more usable versions of the data for research coordinators to work with. Over time data analysts (working with domain experts) may produce additional mapping tables and categorization systems that make it easier for researchers and research co-ordinators to work with the data.
De-identification analysts	Are responsible for ensuring that as many data as possible are made available to research coordinators for the creation of cohorts but that no identifiable data reach the coordinators. Much of the de-identification task is automated, but the system needs to be continually monitored and new DICOM tags added to the whitelist (or blacklist) as required.
System administrators	Are part of the infrastructure team and are responsible for building and maintaining the underpinning infrastructure, security, network separation, monitoring and supporting automated processes. Supported automated processes would involve checking, e.g., whether there were errors in the data load process or data extraction process. They have privileges and expertise to debug and/or restart these processes.
Software developers	Produce any new software required within any zones of the environment. The software is developed and tested outwith the production environment. Deployment of software updates will be carried out by system administrators.

### Architectural support for feedback and enhancement from other sources

The architecture uses a microservices architecture. Individual components (microservices) can be turned on or off as needed and support multi-process execution for linear scaling. The architecture has been designed to support iterative enhancement based on feedback from research outputs generated in the researcher Safe Haven environment or directly from external sources (e.g., clinical experts). Such enhancements could be:

- New datasets, such as clinical mark-up (capturing ground truth data that have been generated by a radiologist marking up data for a set of images)
- New processes to improve cohort generation or dataset preparation (e.g., software that runs over pixel data and returns the size of the airways shown in CT scans), i.e., derived datasets.
- Algorithms that could run over source images or textual data (e.g., software that uses natural language processing on imaging metadata to find images that show signs of dementia).

There are several key benefits:

- This is an opportunity to incrementally improve the quality and value of datasets from SMI.
- Research projects could add expertise at a scale that will never be available within a single development team.
- It can improve collaboration and sharing across projects.
- It supports active engagement by the user community and increases support for the service.

## Analysis

### Current status of the data

A copy of the historical imaging data (from 2006 to September 2018) in PACS proprietary format has been transferred onto the hardware environment and converted into the non-proprietary DICOM format. A feed from the National PACS to retrieve the data from October 2018 onwards is in the process of being commissioned.

The first system test was conducted using ∼3 million studies (∼10 million series and ∼300 million images). These data (all the scans taken during the same 2-week period in February for 7 consecutive years) have been used as test data for software development.

The implementation has enabled extraction of images based on cohorts built from data captured in DICOM tags and linking data from other sources—as illustrated in the “Use Cases” section. At this stage, operation still involves some manual intervention that we intend to automate as development progresses, and only the initial subset of DICOM tags (as recommended by a domain expert) is promoted, but the system is designed to facilitate enhancement and extension in future [18].

### Justification of different tools within the plugin architecture

#### Core platform

The platform has been implemented building upon the open source Research Data Management Platform (RDMP) [19]. The RDMP stores, manages, cleans, de-identifies, and processes data to create reproducible, auditable data extracts for research and in the past 5 years has been used to support >500 projects, generating >2,000 data extracts of mainly phenotypic text-based data for epidemiological research projects and clinical trials. The RDMP already provides many of the core components such as auditing, logging, deduplication, and anonymisation required for populating the relational database in a platform-agnostic way, as well as linkage and extraction; therefore it was efficient to build upon this platform to handle imaging data as well (creating the “**i**maging RDMP” [iRDMP]).

#### Choice of architecture

A microservice architecture using the RabbitMQ message broker [18] simplifies development, testing, and refinement of components in isolation, minimising and containing the adverse effects of changes. A microservice architecture is one that decomposes a monolithic application into a set of smaller loosely coupled services communicating over well-defined interfaces [20]. Advantages of a microservice architecture as opposed to a monolithic approach have been known in the IT industry in recent years (e.g., Amazon [21], Netflix [22]) and recently for health data [23].

#### Non-structured database solution for identifiable metadata

The data in the DICOM tags are largely unstructured and deeply hierarchical. This is challenging to represent in a relational store. Moreover, their structure may change over time (e.g., new tags). Consequently, the use of a document-oriented, flexible, and dynamic data storage system was deemed necessary. MongoDB [24] was selected as the NoSQL database technology because the hierarchy of DICOM tags can be mapped directly to a JSON document, then indexed and queried efficiently. This facilitated the transfer of images across database collections and use of queries against DICOM tags to select and control the promotion of data to later stages in the process.

Within the architecture the MongoDB database provided a middle ground within the ETL data flow. It allows mappings to the relational database schemas to be quickly modified and tested, while being able to quickly reload and reprocess data from MongoDB rather than the slow process of going back to DICOM files, reducing the petabytes of raw DICOM files to terabytes of queryable data.

#### Structured database solution for de-identifiable metadata

Many DICOM servers and APIs have a way of representing DICOM in a relational schema, e.g., dcm4chee [25]. We have used our own (dynamic) cut-down schema for several reasons:

- To present something to data analysts that has a simplicity (without requiring DICOM expertise) on the same level as the other linkable datasets hosted on the National Safe Haven.
- To optimise for linkage, i.e., the ability to limit the number of table joins needed and create efficient query-oriented indexes, e.g., PatientId+ImageType+StudyDescription.
- To be able to adjust this schema and regenerate the data as future development requires.
- To be able to add additional curated fields from external sources or transformed columns such as results from expert mark-up as ground truth data.
- To be able to store (and therefore expose) a limited set of tags (those we understand will not contain identifiable data).

#### Anonymisation tools

There are many different software programs that can be used to de-identify imaging data. We tested the feasibility of 3 different widely used programs (DICOM Confidential [26], XNAT [27, 28], CTP [29]) in deciding which to adopt as part of the pipeline. A summary of each is provided in Online Appendix B.

For a meaningful comparison of the tools, a set of criteria were devised, and each de-identification program was examined in turn against these criteria using a rating of 1–5 (where 5 is the best). We grouped the results into 3 different categories: core functionality, user friendliness, and support. Table 4 provides a summary of the scores for each category, with the detailed analysis provided in Online Appendix A.

**Table 4:** Score of different de-identification tools

Tool	Core functionality	User friendliness	Support	Total
XNAT	37	21	22	80
CTP	41	24	25	90
DICOM Confidential	35	24	14	73

Maximum scores: 45, 30, 25 for a total of 100.

In summary, DICOM Confidential was ruled out owing to the quality of the documentation and the lack of first-party or community support. We found that some of the images produced by DICOM Confidential were corrupted and chose not to investigate any further because the functionality of the other 2 tools seemed superior.

There was little difference in the functionality of CTP and XNAT. They are both well-supported tools that could perform the required tasks. The overall score of CTP was higher than XNAT. We thought that the XNAT image “bundling” for applying rules to subsets of images would be a useful capability that CTP does not provide. The pixel-level anonymisation capability seemed to be much better supported and straightforward in CTP, and this is very important for this project. For these reasons we chose CTP.

#### NIFTI as a method of de-identification

NIFTI (Neuroimaging Informatics Technology Initiative) is an alternative to DICOM as a medical image storage file format. Originally created for neuroimaging, NIFTI stores image data as a single 3D image (.nii file), whereas DICOM stores a separate image file for each slice of the scan. In addition, the NIFTI format only stores pixel data and metadata related to the image itself, not any patient or study information as one would find in a DICOM image. This makes NIFTI a possible method to “anonymise” DICOM images. Not all image modalities and compression methods are supported however, and conversion tools require extensions to interpret the private tags that some image scanners write into the DICOM files to describe the pixel data. Therefore, NIFTI was not chosen.

NIFTI has become popular in some machine learning applications and is preferred over DICOM owing to the ease of dealing with only 1 file representing the whole 3D scan. The images for each research project can be provided in a range of formats (including NIFTI), but conversion to NIFTI was not adopted as a method for de-identification.

#### Pixel data and anonymisation

Primary original CT scans were found to contain no “burned-in” text, i.e., no text within the pixel data. For MRI and other images we integrated a text detection tool into our extraction pipeline, running each image through the Tesseract open source OCR tool (originally developed by Hewlett-Packard) to detect the presence of any readable text.

In some cases, particularly head scans, the pixel data themselves may be inherently identifiable [30–32]. This requires special-purpose software, however, because the pixel data are never exported beyond the Safe Haven environment and the use or installation of such software would not be permitted, and any research project would be denied access to the data for this purpose. In a very few cases something could be identifiable by unaided inspection—e.g., a distinctive injury or piece of jewellery; this is an issue that needs further consideration.

#### Software deployed in the Safe Haven analytical environment

We investigated several tools to deploy into the Safe Haven for managing, viewing, and manual annotation of images by research teams. We chose MicroDICOM (simple DICOM viewer) [33] as the first example to use. Over time it is expected that the number of tools available as part of the pre-installed VM will increase and that researchers will have the capability to install their own preferred software tools.

### Comparisons to other existing systems

Given the different imaging platforms in active development to support research projects, we investigated alternative platforms so that we did not re-invent the wheel. In general terms, other solutions have concentrated on consented cohorts from researcher-collected research images rather than much larger unconsented data from routinely collected “real-world” images. The architectural solution developed by others is generally a large anonymised database (sometimes distributed) containing all the images with permissions to see, extract, and run pipelines on the imaging data configured for each research group. The metadata provided are limited and relatively clean in comparison with routinely collected data. The architectural challenges and solutions are therefore very different. For example, a key functionality of the platform is the efficient and effective selection of anonymised cohorts from petabytes of noisy and heterogeneous identifiable data.

If the requirements were to store a de-identifiable, clean, homogenized copy of all of the pixel data and metadata within the de-identifiable zone, we could have used one of the many excellent open source platforms for managing large volumes of imaging data such as OMERO [34, 35], XNAT [27, 28], or ClearCanvas [36]. There are several reasons why we did not choose this approach and therefore did not use such platforms to manage the core data repository:

- We envisage that the methods to de-identify data will change over time as our understanding increases and technological solutions improve. It is impractical to re-create >2 PB of de-identified images each time our methodology improves.
- It is unnecessary to undertake the effort to validate any de-identification method on all DICOM tags when only a small fraction of these will be required by research teams. It is unknown which ones will be required up front.
- A proportion of all the images will never be extracted/released for research projects because they will not meet the cohort requirements. De-identifying imaging data reactively, only when required for a specific project, removes the requirement to carry out a needless time-consuming and computationally expensive de-identification process on images that are never required. (Conversely, a given image may be de-identified multiple times, once for each project. This is an issue that we plan to resolve in the next stage of development. We expand on this in the Discussion section).
- It is risky to test a specific de-identification tool on sample data and trust that it will therefore also be successful for variations of routinely collected data from multiple sources and vendors. The architecture was designed to reduce this risk by default blacklisting all data until proven otherwise, in which case the metadata and/or image is then “promoted” to a whitelist.
- The data are currently >2 PB and expected to grow at a rate of ∼400 TB per year. There is substantial cost in maintaining 2 copies of the data both in terms of hardware and the maintenance required to update a duplicate as new data arrive (an identifiable version of the data is required in the identifiable zone to meet Requirement 1—see Online Appendix A).
- Hosting duplicate versions of the data introduces additional data security and governance risks.
- Different research projects require different de-identification. For example, the granularity of date and patient age data may change depending on the specific questions posed by a research project; the overarching rule is that the data be de-identified as far as possible while meeting the research requirement.
- Following the data protection principle that individuals should see the minimum data to fulfil their job role, there is no need for research co-ordinators to see the pixel-level data to build cohorts; therefore, only text-based metadata are provided for cohort building.

Although existing solutions will not fully meet the requirements of this programme, one of our core principles is to reuse as many applicable, open source or freely available tools as possible; i.e., do not try to re-invent the wheel. Therefore, where relevant, we have included other software within our architecture.

### Testing

The SMI microservices, and the RDMP framework upon which it relies, have been developed entirely using a test-driven development approach. Continuous integration (CI) unit and system integration tests ensure code stability. Approximately 1,450 automated tests cover the core RDMP code base, and in excess of 300 tests run on the SMI microservices.

Following development of a baseline version, functional and non-functional manual testing was undertaken. The test cases were planned and documented in advance, following a series of interviews with clinicians, academics, and technical staff. While these scenarios were planned, documented, and agreed upon, the approach to executing the tests was deliberately as exploratory as possible rather than restricted by specific test scripting.

#### Exemplar-driven use cases

A number of use case scenarios were defined with input from researchers (listing in Online Appendix D). These scenarios were further elaborated by a team from eDRIS, in effect, performing a dry run as though these were real research projects. The test cases assessed included the following scenarios:

- Cohorts can be generated using the metadata repository information
- Images can be returned where the cohort has been generated from another dataset
- Cohorts and images can be identified using a combination of the metadata repository and other data sources

#### Scalability testing

Performance was benchmarked at the main processing stages of the end-to-end solution: initial load, population of the relational DB, and extraction/anonymisation.

##### Initial Data Load: DICOM files to MongoDB

- Speed of population: 66 days per year of data
- Disk space consumed: 7.2 kB per image, 2 TB per year of data

##### Populating the metadata repository

- Speed of load: ∼600 image rows per second; 6 days per year of data
- Disk space consumed: 180 GB per year of data

Once deployed to the National Safe Haven and tuned, performance proved sufficient to ingest the initial snapshot of data (up to September 2018) and is expected to keep up with future data feeds from the PACS once that is provisioned.

### Extraction and anonymisation

Test were run on increasing numbers of images, and the processing time was logged as shown in Table 5. These tests were run using CT scans only (other modalities can have a significantly higher number of images per series and/or larger file sizes). Each DICOM file is ∼0.5 MB. On average each CT series has 325 images, giving a total file size of ∼170 MB. The hardware on which this runs is summarized in Online Appendix C.

**Table 5:** Scalability of anonymisation processing

No. image IDs	Anonymisation run time	Total file size	Mean run time per file
3	Negligible	1.5 MB	Negligible
2,264	0.3 hours	1.2 GB	0.54 sec
89,415	1.5 hours	50 GB	0.06 sec
∼1.2 million	28 hours	630 GB	0.06 sec
∼17.2 million	65 hours	8.5 TB	0.01 sec

## Discussion

The system we have developed is not a new tool for managing and viewing images like XNAT, OMERO, MicroDICOM, and ClearCanvas. iRDMP is a platform and pipeline for extracting images from a directory of images based upon cohort selection criteria, anonymising them and copying the images into a secure location for analysis. Theoretically a tool/system for managing and viewing images from a single data store could have been configured/enhanced with a permissions layer to restrict access to only the images each research group had the right to see. This model was discounted because it did not meet the requirements for several reasons:

- Risk of hacking
- Risk of de-identification going wrong
- Speed of access
- Researchers wishing to use their favourite tools to manage and manipulate imaging data
- Cohort building functionality and linking to other datasets unavailable within such tools
- Speed of de-identification on the fly, or needing 2 copies of all of the data

### Applicability/potential of the architecture and platform to be utilized in other environments or use cases

There are many different platforms in active development to support multiple research projects using clinical imaging data. Our architecture has not just been designed to fulfil Scottish data governance principles and data structures—there is a much wider applicability. There are many other Safe Havens nationally and internationally [37, 38] where such a solution might be applicable, and there is a trend towards the creation of new Safe Havens. Although within our architecture data extracts are viewed within a Safe Haven Analytical Platform (as part of the Scottish Data Governance requirements), the software platform can extract data to any destination. The software could therefore be utilized by other groups or organizations to manage imaging data and build cohorts for extraction that do not use Safe Haven environments.

We have tested our software on 2 different environments with different hardware and VM tools: a regional Safe Haven and the National Safe Haven. It proved flexible enough to work in both environments.

We have created a Docker-based integration repository [39] that supports automated testing (in Travis CI) of the full stack of microservices with test data generated by BadMedicine.Dicom (BadMedicine, RRID:SCR_018879) [40]. This ensures that deployment of the tech stack is simple and reproducible.

### Potential impact of enabling this resource

The SMI data, linked to other datasets, along with the secure iRDMP platform we have developed have the potential to reduce the costs and widen access to large quantities of routinely collected de-identified images at scale. They also have the potential to reduce the effort of obtaining governance approval because a data controller–approved method for de-identification and access has already been agreed. Increasing the availability of large-scale routinely collected imaging datasets linked to other forms of health data for both industry and academic use will hopefully lead to a greater likelihood of achieving results translatable into diagnoses and treatments.

### Future plans

#### Short term

There are several developments that will enhance the functionality beyond that already provided, which we aim to implement in the near future. Rather than the limited subset of CT and MRI metadata tags currently promoted, we plan on promoting many more of these tags. We would like to trial the use of the wider RDMP tool for cohort building and audit within the de-identifiable zone. This will require training on the tool and slight modifications to existing workflows. We would also like to fully automate the processes once the testing of the components has been completed. We are in the process of loading all historical data into the system, after which time we would like to carry out some performance testing of the solution to identify and investigate bottlenecks.

#### Medium term

As well as enabling other modalities (in addition to CT and MRI), we would like to support complex cohort building:

- **Structured Reports** are summary information mainly stored in free-text format that have been populated by a clinician about the study. They can include patient information such as why the scan was requested in the first place, the condition found, and family history. A cohort derived from structured reports might seek to extract all the images where a CT scan was performed because a lung tumour was expected. Structured reports are challenging to query because they can be highly identifiable and are free text and sparsely populated. As such, natural language processing methods have been widely used to extract information from the reports. We plan on utilising and extending many of these methods within the platform to extract relevant metadata from the reports that can then be used for complex cohort building.
- **Pixel Data** contain information that could be helpful for building a cohort of relevant images, e.g., extracting all x-ray images of the knee where the depth of cartilage is <2 mm. This information is not captured in the DICOM metadata and instead would be obtained using an image-processing algorithm to extract supporting features. We plan on developing automation processes where potentially relevant images are opened and the algorithm applied to the pixel data returning the cartilage depth. The cartilage depth can then be used to link with other data.
- **Complex DICOM Metadata:** The same information can be found within different DICOM tags depending on the source; e.g., identifying an image as a susceptibility-weighted imaging sequence requires checking 3 different fields for the occurrence of 1 of 4 possible strings and then filtering out some specific mismatches. This leads to problems of standardization, metadata, and definition of data dictionaries. In Scotland there are 4 different radiology information systems that hold data in different ways. There are additional complexities due to conflicting requirements for standardization for the purposes of cohort building and research use. We plan to develop algorithms for text mining and imaging metadata standardisation to provide summary data (data dimensioning), which can then be logically queried for cohort selection. We plan on investigating unsupervised machine learning techniques to group images into commonly used clusters such as body area.

#### Long term

Simply copying pixel data for each research project may not scale for imaging data, where storage could quickly become infeasible as the Safe Haven hosts ever greater numbers of studies each requiring large imaging datasets. An efficient method of sharing the pixel data between multiple studies may be required. However, each study will have different metadata, e.g., study-specific patient identifiers in the image header, so a solution that combines shared pixel data with study-specific non-pixel metadata is needed. We plan to investigate different solutions such as a virtual file server (already developed in prototype), requiring each research group to purchase more disk space should their project require it, pulling images in batches/caching or another technical solution entirely. Different strategies for serving images may be required, such as a file share for machine learning consumption but a DICOM server when using a DICOM image viewer.

We are fortunate to have received significant funding from the Medical Research Council (MRC) and Engineering and Physical Sciences Research Council (EPSRC) to deliver all these future plans within a 5-year programme grant called PICTURES (Interdisciplinary Collaboration for Efficient and Effective Use of Clinical Images in Big Data Health Care Research).

We are very interested in collaborating with other groups working on any of these issues.

## Limitations of the Architecture

We are aware of some limitations of the current architecture:

- Data quality is an issue inherent in the reuse of routinely collected clinical data (as opposed to data being collected specifically for research purposes), e.g., typographical errors marking an MRI as a “Brian” scan rather than “brain”—something overlooked as irrelevant for clinical use, but needing extra attention here.
- The “unconsented” nature of these data mandates control over the research data provided to projects to guard patient privacy, limiting the options for such projects; research is ongoing to mitigate this.
- Automatic detection and redaction of text is essential on this scale but still needs manual intervention and tuning to keep redaction to a low enough level to deliver useful data. To date, 869 “special case” rules have been added to the IsIdentifiable tool's dataset: e.g., the “Princess Royal” hospital being identified as a name rather than an organization.
- Huge number of images can make cohort creation cumbersome at the image level. To address this, we are adding support to the infrastructure and relational database to enable research coordinators to mostly operate at the study level.
- By not anonymising each image once on initial receipt we introduce additional complexity and increased storage requirements. In addition, we have to repeat the anonymisation task each time the image is used, so we have more work to do. We trade this off against the ability to apply better anonymisation later. If the repetition proves an issue in real use, this can be mitigated through caching previously processed pixel data.
- Duplicating the image data for each research project will limit future scaling to multiple projects; some potential ways of addressing this are discussed in the previous section.
- The relational database structure is not ideal for some more complex parts of DICOM. We believe that for cohort generation our flattened relational structure is simple and functional, but we may discover cases in the future where it becomes cumbersome for some parts of DICOM. If so, hierarchical data can be incorporated within MySQL via JSON columns.

## Conclusions

We have designed an architecture that meets the requirements of data governance and security, and initial indications suggest that it will manage and provide extracts of routinely collected imaging data linked to other relevant datasets for research from the >2 PB of SMI data. We have tested the extraction system on 5 use cases, based on real exemplar scenarios.

The Background section of this article identified 5 challenges to using routinely collected clinical images for research.

The limitations of existing patient-centric image handling, Challenge 1, are addressed by extracting and indexing other attributes identified by researchers: instead of retrieving a specific patient's imagery, we can search for images by a combination of parameters such as body part, patient age, or cross-referencing with other datasets, e.g., “all head/brain MRIs of patients with a diagnosis of glioblastoma.”

To address Challenge 2, the pervasive presence of potentially identifying information within various data fields and the image pixels themselves, the platform uses a data controller–approved, standardized de-identification workflow, with a “defence in depth” strategy employed: even if the anonymisation procedures fail earlier in the pipeline, unexpected information is detected and reported to the system operators before release to researchers. To mitigate the risks to the data controllers of providing unconsented routinely collected images for research the platform works in accordance with the Scottish Government guiding principles for secure linking, anonymising, and analysing datasets for research, where a subset of data for a specific cohort are linked for an approved research project and access provided via a Safe Haven environment (the NHS Scotland term for a Trusted Research Environment) [41]. Access to the data can be revoked by the data controller at any time, and researchers cannot extract/output any information other than aggregate-level results from the environment. There is a separation of the roles of indexer, linker (carried out by a trusted third party or Safe Haven staff), and researcher.

The original identifiable data are maintained to enable linkage to other datasets (addressing Challenge 3, the barrier that anonymisation normally presents to such linking) but are not released for cohort building or for research. The use of encrypted numerical patient identifiers (using the same encryption for both image metadata and other eDRIS datasets) also facilitates some linkage without exposing the original identifier: an MRI scan can be matched with a cancer diagnosis or patient admission record without exposing that patient ID.

Developing the handling, indexing, and anonymisation system in a robust, reusable way and incorporating multi-layered safeguards allays data controller concerns about the quality of anonymisation (Challenge 4), while amortising the substantial development and testing costs across multiple research projects makes it more cost-effective to extract large sets of images for deep learning purposes, which might otherwise be uneconomical (Challenge 5).

No non-trivial dataset is likely to be perfect, particularly when data gathered for one purpose is being re-purposed, so each research project will need to apply appropriate quality control checks; some will also bring new value, e.g., an expert analysis of the images for a specific purpose. Each time an issue is identified by one project or new information added, this has the potential to improve the resources available to future projects. In the absence of a pre-created perfect reference library, any project will have to choose between using a resource such as this, with the need for quality checks, and a much smaller but more discerning research-specific set if one is available or could be created. With CT scans in particular, the radiation dosage makes the reuse of existing imagery much more feasible.

To access the SMI dataset for a research project, please contact eDRIS [17] in the first instance.

## Availability of Source Code and Requirements

Snapshots of our code and other supporting data are openly available in the *GigaScience* repository, GigaDB: https://dx.doi.org/10.5524/100780

**Project name**: Research Data Management Platform

**Project home page**: https://github.com/HicServices/RDMP

**Registration**: biotools:rdmp RRID:SCR_016268

**Synthetic data generation (used in integration testing):**

https://github.com/HicServices/BadMedicine (EHR,

biotools:badmedicine, RRID:SCR_018879)

https://github.com/HicServices/BadMedicine.Dicom

(DICOM data component for the above)

**Template building and relational database loading library:**

https://github.com/HicServices/DicomTypeTranslation

(translation between DICOM types and database types,

also includes example modality templates: biotools:

dicomtypetranslation, RRID:SCR_018878)

https://github.com/HicServices/DicomTemplateBuilder

(GUI editor for building templates)

**Imaging Repos**itories

https://github.com/HicServices/RdmpDicom (RDMP plugin

for imaging)

https://github.com/SMI/SmiServices (imaging microservices,

biotools:smi_services, RRID:SCR_018881)

https://github.com/SMI/Integration (full stack testing for

microservices)

Operating system(s): Windows (GUI) or Linux (console)

Programming language: C#

Other requirements: Microsoft SQL Server

License: GPL v3

No restrictions to use by non-academics.

## Availability of Supporting Data and Materials

Other data, including links to additional information, further supporting this work can be found in the *GigaScience* repository, GigaDB [42].

## Additional Files

Appendix A: Summary of requirements

Appendix B: Analysis of anonymisation tools

Appendix C: Summary of the hardware infrastructure

Appendix D: Use cases

Supplementary Table 1: Core functionality

Supplementary Table 2: User friendliness

Supplementary Table 3: Support

## Abbreviations

CHI: Community Health Index; CT: computed tomography; DB: Database; eDRIS: Electronic Data Research and Innovation Service; EHR: Electronic Health Record; EPSRC: Engineering and Physical Sciences Research Council; ETL: Extract Transform Load; EUPI: Encrypted Universal Patient Identifier; GUI: graphical user interface; HIC: Health Informatics Centre; ISD: Information Services Division; JSON: Javascript Object Notation; MRC: Medical Research Council; MRI: magnetic resonance imaging; NHS: National Health Service; NIFTI: Neuroimaging Informatics Technology Initiative; OCR: optical character recognition; PACS: Picture Archive Communication System; PI: principal investigator; RDMP: Research Data Management Platform; SMI: Scottish Medical Imaging; SQL: Structured Query Language; VM: virtual machine.

## Competing Interests

The authors declare that they have no competing interests.

## Funding

The authors acknowledge the support from the Farr Institute of Health Informatics Research and Dundee University Medical School. This work was supported by the Medical Research Council (MRC) grant No. MR/M501633/1 (PI: Andrew Morris) and the Wellcome Trust grant No. WT086113 through the Scottish Health Informatics Programme (SHIP) (PI: Andrew Morris). SHIP is a collaboration between the Universities of Aberdeen, Dundee, Edinburgh, Glasgow, and St Andrews, and the Information Services Division of NHS Scotland. This project has also been supported by MRC and EPSRC (grant No. MR/S010351/1) and by the Chief Scientist Office of the Scottish Government Health and Social Care Directorates via a leverage grant made to Farr Scotland. The project was also supported by the Scottish Government through the “Imaging AI” grant award.

This work was supported by Health Data Research UK, which receives its funding from HDR UK Ltd (HDR-5012) funded by the UK MRC, Engineering and Physical Sciences Research Council, Economic and Social Research Council, Department of Health and Social Care (England), Chief Scientist Office of the Scottish Government Health and Social Care Directorates, Health and Social Care Research and Development Division (Welsh Government), Public Health Agency (Northern Ireland), British Heart Foundation (BHF), and the Wellcome Trust

## Authors’ Contributions

E.J. conceived, led, and directed the project and drafted this manuscript. G.M., T.N., M.A., and A.H. designed the architecture. T.N., L.T., G.M., A.H., R.M., J.S., A.B., B.P., and R.T. all developed and tested the software. C.S., C.M., J.C., and I.B. all provided requirements for the system, supported the testing of the software, and provided data governance support and expertise. D.S.-M. and J.R.H. provided database development and support. D.S., W.K., J.W., K.G., and D.M. provided operational support and development for the underpinning Safe Haven infrastructure. A.M. and C.S. chaired and provided expertise as part of the project oversight group. A.M. and M.P. provided leadership for this project as part of the wider Farr and HDR UK initiatives. R.B., S.K., and D.H. were project managers. All authors read, edited, and approved the final manuscript.

## Supplementary Material

giaa095_GIGA-D-20-00076_Original_Submission

giaa095_GIGA-D-20-00076_Revision_1

giaa095_GIGA-D-20-00076_Revision_2

giaa095_Response_to_Reviewer_Comments_Original_Submission

giaa095_Response_to_Reviewer_Comments_Revision_1

giaa095_Reviewer_1_Report_Original_Submission Renato Cuocolo -- 4/16/2020 Reviewed

giaa095_Reviewer_1_Report_Revision_1 Renato Cuocolo -- 7/30/2020 Reviewed

giaa095_Reviewer_2_Report_Original_Submission Jim Davies -- 5/20/2020 Reviewed

giaa095_Supplemental_File
