# Supplementary material for: An extensible big data software architecture managing a research resource of real-world clinical radiology data linked to other health data from the whole Scottish population
Source: Gigascience. 2020 Sep 29;9(10):giaa095. doi: 10.1093/gigascience/giaa095 (PMC7523405; doi:10.1093/gigascience/giaa095)
Supplement: giaa095_GIGA-D-20-00076_Original_Submission [file giaa095_giga-d-20-00076_original_submission.pdf]

GigaScience

An extensible big data software architecture managing a research resource of real-world clinical radiology data linked to other health data from the whole Scottish Population

--Manuscript Draft--

**Manuscript Number:**	GIGA-D-20-00076
**Full Title:**	An extensible big data software architecture managing a research resource of real-world clinical radiology data linked to other health data from the whole Scottish Population
**Article Type:**	Technical Note
**Funding Information:**	Medical Research Foundation (MR/M501633/1) — Prof Andrew Morris<br>Wellcome Trust (WT086113) — Prof Andrew Morris<br>Medical Research Foundation (MR/S010351/1) — Prof Emily Jefferson<br>Chief Scientist Office (Leverage grant made to Farr Scotland) — Prof Andrew Morris<br>Scottish Government (Imaging AI Project) — Prof Emily Jefferson<br>Health Data Research UK (HDR-5012) — Dr Mark Parsons
**Abstract:**	Aim: To enable a world-leading research dataset of routinely collected clinical images linked to other routinely collected data from the whole Scottish National population. This includes 30 million different radiological examinations from a population of 5.4 million and over 2 petabytes of data collected since 2010. Methods: Scotland has a central archive of radiological data used to directly provide clinical care to patients. We have developed an architecture and platform to securely extract a copy of that data, link it to other clinical or social data sets, remove personal data to protect privacy, and make the resulting data available to researchers in a controlled Safe Haven environment. Results: An extensive software platform has been developed to host, extract and link data from cohorts to answer research questions. The platform has been tested on 5 different test cases and is currently being further enhanced to support 3 exemplar research projects. Conclusions: The data available is from a range of radiological modalities, scanner types and collected under different environmental conditions. This “real-world”, heterogenous data is highly valuable for training algorithms to support clinical decision making, especially for deep learning where large data volumes are required. The resource is now available for international research access. The platform and data can support new health research using Artificial Intelligence and Machine Learning technologies as well as enabling discovery science.
**Corresponding Author:**	Emily Jefferson, PhD<br>University of Dundee School of Medicine<br>UNITED KINGDOM
**Corresponding Author Secondary Information:**	
**Corresponding Author's Institution:**	University of Dundee School of Medicine
**Corresponding Author's Secondary Institution:**	
**First Author:**	Thomas Nind, PhD
**First Author Secondary Information:**	
**Order of Authors:**	Thomas Nind, PhD<br>Gordon McAllister, PhD<br>Douglas Hardy

Powered by Editorial Manager® and ProduXion Manager® from Aries Systems Corporation

	Ally Hume, PhD
	Ruairidh MacLeod, PhD
	Jacqueline Caldwell
	Leandro Tramma
	Ross Teviotdale
	Mohammed Abdelatif
	Kenny Gillian
	Joe Ward, PhD
	James Sutherland, PhD
	Donald Scobbie
	Andrew Brooks, PhD
	Bianca Prodan
	William Kerr
	Dominic Sloan-Murphy
	Juan Rodriguez Herrera
	Dan McManus
	Carole Morris
	Carol Sinclair
	Rob Baxter, PhD
	Mark Parsons, PhD
	Andrew Morris, CBE MD FRCP FRSE FMedSci
	Emily Jefferson, PhD
**Order of Authors Secondary Information:**	
**Additional Information:**	
**Question**	**Response**
Are you submitting this manuscript to a special series or article collection?	No
**Experimental design and statistics**<br><br>Full details of the experimental design and statistical methods used should be given in the Methods section, as detailed in our Minimum Standards Reporting Checklist. Information essential to interpreting the data presented should be made available in the figure legends.<br><br>Have you included all the information requested in your manuscript?	Yes
**Resources**	Yes

A description of all resources used, including antibodies, cell lines, animals and software tools, with enough information to allow them to be uniquely identified, should be included in the Methods section. Authors are strongly encouraged to cite Research Resource Identifiers (RRIDs) for antibodies, model organisms and tools, where possible.<br><br>Have you included the information requested as detailed in our Minimum Standards Reporting Checklist?	
**Availability of data and materials**<br><br>All datasets and code on which the conclusions of the paper rely must be either included in your submission or deposited in publicly available repositories (where available and ethically appropriate), referencing such data using a unique identifier in the references and in the “Availability of Data and Materials” section of your manuscript.<br><br>Have you have met the above requirement as detailed in our Minimum Standards Reporting Checklist?	Yes

Powered by Editorial Manager® and ProduXion Manager® from Aries Systems Corporation

Manuscript Click here to download Manuscript iRDMP Architecture No Figures or Tables.pdf

An extensible big data software architecture managing a research resource of real-world clinical radiology data linked to other health data from the whole Scottish Population

Abstract

Aim: To enable a world-leading research dataset of routinely collected clinical images linked to other routinely collected data from the whole Scottish National population. This includes 30 million different radiological examinations from a population of 5.4 million and over 2 petabytes of data collected since 2010.

Methods: Scotland has a central archive of radiological data used to directly provide clinical care to patients. We have developed an architecture and platform to securely extract a copy of that data, link it to other clinical or social data sets, remove personal data to protect privacy, and make the resulting data available to researchers in a controlled Safe Haven environment.

Results: An extensive software platform has been developed to host, extract and link data from cohorts to answer research questions.

The platform has been tested on 5 different test cases and is currently being further enhanced to support 3 exemplar research projects.

Conclusions: The data available is from a range of radiological modalities, scanner types and collected under different environmental conditions. This “real-world”, heterogenous data is highly valuable for training algorithms to support clinical decision making, especially for deep learning where large data volumes are required. The resource is now available for international research access.

The platform and data can support new health research using Artificial Intelligence and Machine Learning technologies as well as enabling discovery science.

Background

Advantages and challenges of using routinely collected clinical images for research

Clinical images, especially when linked to other routinely collected health data, are extremely useful for many types of research: examining early/preclinical diagnosis [1], disease progression [2, 3], genotype-phenotype associations [4], development of risk profiles [5, 6], computer vision methods for biomarker extraction [7, 8], machine learning approaches [9], and discovery and classification of disease types [10]. The emerging field of Radiomics has the potential to bridge the gap between medical imaging and personalised medicine [11]. However, collecting images for specific research projects is expensive and constrains the scale of many studies. Research cohorts are usually comprised of a narrow subset of people with a condition, which can make both generalising findings and repurposing of images for research problematic. Use of routinely collected images, in contrast, opens up the potential for very large-scale studies, which not only efficiently and effectively complement smaller disease-based cohorts of patients but are also extremely flexible when linked to extensive electronic medical records allowing for a wide range of disease areas to be examined. However, whereas research images are typically collected using specific image acquisition protocols under ideal conditions, routinely collected clinical images are much more heterogeneous.

Using clinical images for research and linking them to other routinely collected clinical data is challenging because:

1) Existing software used to query/search for images from PACS (Picture Archive Communication System) is designed for clinical care rather than research. They make it easy to find all images for a particular patient, but they are not designed to facilitate searching for all images with particular characteristics such as body part, slice thickness/scanning protocol/contrast agent/patient medication or linking to other EHR datasets (such as outcome data, prescription data).
2) Reuse of clinical images for research requires de-identification, yet identifiable data can be present in many areas of the associated DICOM (Digital Imaging and Communications in Medicine)[12] file metadata and/or may be present within the pixel data itself, 'burned on' to the actual image.
3) Anonymisation of images can reduce the ability to perform linkage to other datasets e.g. demography, prescribing, hospital admissions etc.
4) Reuse often requires approval from multiple Data Controllers, and the complexity of de-identification increases the risk of rejection of applications for research given the amount of work the Data Controller may have to do to ensure that no identifiable data is released.
5) For deep learning projects, where large numbers of images are required, the image extraction costs for research can be prohibitive.

Scottish Clinical and Research Data

Scottish Clinical PACS System: Scotland has a single National PACS Clinical System which contains all radiological images collected from 14 different health boards. To date (2019), this includes 30 million different radiological examinations from a population of 5.6 million and over 2 petabytes of data collected since 2010. It includes a range of modalities (including computed tomography (CT), magnetic resonance imaging (MRI), ultrasound, nuclear medicine imaging and plain film radiography). This system is a live environment which is used directly for clinical care.

Provision of routinely collected text-based clinical data for research: Scotland has a relatively stable population with long-established use of a unique healthcare identifier (the Community Health Index [CHI] number) that is also increasingly seeded in data in other sectors such as social care. An National Health Service (NHS) Scotland service, called the electronic Data Research and Innovation Service (eDRIS) [13], provides a National Safe Haven environment (hosted by the University of Edinburgh) to support research access to anonymised extracts of linked data from different Data Controllers to answer specific approved research questions. The linkable phenotypic data includes a range of national datasets including, for example, prescribing, death data and hospital admissions.

Incorporating clinical imaging data into the wealth of available datasets for research: A research copy of the data held within the Scottish National Clinical PACS system has been created to enable the clinical imaging data to be linked with the other routinely collected datasets and be made accessible for research (given appropriate data governance approvals). The research copy of the Clinical PACS system is called the Scottish Medical Imaging (**SMI**) Database and the data is held in the non-proprietary DICOM format.

The management of imaging data for research presents a substantial set of challenges beyond those encountered in the management of purely text-based records. Some of these are variations on familiar challenges, such as de-identification, whilst others are novel and intrinsic to this type of dataset, such as size and compute requirements for big data processing.

This paper describes the architectural solution and software platform developed to support hosting, extracting and linking the SMI data which addresses the challenges identified of using routinely collected imaging data for research listed above.

We first describe the project approach, a very high level summary of the requirements, then our architectural solution, an explanation of why this solution met the requirements and why our solution is different to that of other open-source solutions for the large scale hosting of imaging data. We explain how the architecture enables feedback and enhancement improvements from other sources.

We then describe our progress towards implementing the architecture and the use cases we have tested.

Project Approach

There have been 4 phases to the project to date:

Requirements gathering: An initial requirements gathering exercise was undertaken at the project inception elucidating requirements from the research community who will use the data extracts provided by the platform, the National Health Service Data Governance representatives as the Data Controllers of the data, and the National Safe Haven staff who will use the platform to build cohorts and provision relevant data extracts to researchers for analysis. We also investigated other open source and freely available platforms for hosting and/or anonymising imaging data to see if any of these could be used entirely or in part within our solution. We researched both functional and non-functional requirements of the solution.

Development of the Architecture: We developed a range of option appraisals and designed an architectural solution to meet the requirements.

Development of Prototype: We developed prototype software to run in a Regional Safe Haven environment managed by the University of Dundee, whilst the SMI data transfer project was taking place in parallel. This prototype supported two consented research projects, predicting dementia from CT and MRI images. We then expanded the prototype to run in the National Safe Haven.

Testing and Case Studies on Sample Data: The software was then tested on a 180TB subset of the full dataset including ~3 million studies (which were loaded into the Document Store - see below). These were images generated across Scotland during the same 2-week period in February for each year in a 7-year period. Linkage, extraction and anonymisation was performed for a range of case studies along with performance and functional testing. The full set of historical data was still in the process of being decrypted from the proprietary PACS vendor format and could therefore not be used for complete testing at this stage.

Platform Architecture

A list of high-level platform requirements is provided in Appendix A.

Architecture Overview

The high-level platform architecture is shown in Figure 1. The Discussion section describes our future plans.

The SMI Data Repository is divided into identifiable and de-identified zones. The SMI Analytic Platform is the Safe Haven environment where researchers can access their relevant data extracts. Figure 1 - Overview of Architecture

Each zone has audited, controlled access with a clear separation of roles and functions. Only System Administrators can access the identifiable zone to carry out maintenance and security functions. Only De-identification Analysts can view the potentially identifiable data in their duties. Research Coordinators can query de-identified text-based (including DICOM tag values) data for cohort building, linkage and extraction in the de-identified zone. Researchers can access de-identified (using Project-IDs) metadata and de-identified pixel data for their cohort within the Virtual Machine (VM) Safe Haven environment.

The CHI number encodes potentially identifiable information such as data of birth, so we use an encrypted version known as EUPI (Encrypted Universal Patient Identifier) within the de-identified zone and this is typically used for patient linking tasks.

Two processes (historic and ongoing) extract DICOM files from the proprietary PACS format received from the NHS. These **DICOM files** contain identifiable metadata (DICOM Tags) which are read and stored in full in the Document Store (a MongoDB database) by the Loader. The DICOM files also contain the pixel image information.

The **Identifiable DICOM Tag Data** is analysed for potentially identifiable data. Relative file paths and a subset of DICOM Metadata which can be de-identified and is useful for cohort building are sent to the **Inventory Tables** (a relational database) and stored using EUPIs within the Identifiable Zone. A mirror of the Inventory Tables is provided to Data Analysts in the De-Identified Zone. This mirror supports condensing tag data e.g. to Study / Series level to improve performance of cohort generation / linkage.

The Inventory Tables can be queried like any other database by Research Co-coordinators to construct cohorts using their existing working procedures. Once a research extract has been created, the corresponding image UIDs (Study / Series or SOPInstanceUID) are supplied to the Extraction process. The extraction process uses the Inventory Tables to locate and anonymise the DICOM files held in the Data Store. These anonymised files are then provided to the researcher in the Safe Haven Environment.

A summary of the expected functionality of the data stores and process within the architecture is provided in Tables 1 and 2.

Table 3: Roles

Architectural support for feedback and enhancement from other sources

The architecture uses a microservices architecture. Individual components (microservices) can be turned on/off as needed and support multi process execution for linear scaling. The architecture has been designed to support iterative enhancement based on feedback from research outputs generated in the Researcher Safe Haven Environment or directly from external sources (e.g. clinical experts). Such enhancements could be:

- New datasets, such as clinical mark-up (capturing ground truth data which has been generated by a radiologist marking up data for a set of images)
- New processes to improve cohort generation or data set preparation (e.g. software which runs over pixel data and returns the size of the airways shown in CT scans) i.e. derived datasets.
- Algorithms which could run over source images or textual data (e.g. software which uses natural language processing on imaging metadata to find images which show signs of dementia).

There are several key benefits:

- This is an opportunity to incrementally improve the quality and value of data sets from SMI.
- Research projects could add expertise at a scale which will never be available within a single development team.
- It can improve collaboration and sharing across projects.
- It supports active engagement by the user community and increases support for the service.

Analysis

Current status

Data

A copy of the historical imaging data (from 2006 to Sept 2018) in PACS proprietary format has been transferred onto the hardware environment. There is an on-going process to convert this historical data into the non-proprietary DICOM format, with expected conversion by May 2020. A feed from the National PACS to retrieve the data from Oct 2018 onwards is in the process of being commissioned.

The first system test was conducted using ~3 million studies (~10 million series and ~300 million images). This data (all the scans taken during the same 2-week period in February for 7 concurrent years) has been used as test data for software development.

The implementation has enabled extraction of images based on cohorts built from data captured in DICOM tags and linking data from other sources – as illustrated in the "Use Cases" section. At this stage, operation still involves some manual intervention we intend to automate as development progresses, and only the initial subset of DICOM tags (as recommended by a domain expert) is promoted, but the system is designed to facilitate enhancement and extension in future. [14],

Justification of different tools within the plugin architecture

Core platform

The platform has been implemented building upon the open source Research Data Management Platform (**RDMP**)[15]. The RDMP stores, manages, cleans, de-identifies and processes data to create reproducible, auditable data extracts for research and in the last 5 years has been used to support over 500 projects, generating over 2000 data extracts of mainly phenotypic text-based data for epidemiological research projects and clinical trials. The RDMP already provides many of the core components such as auditing, logging, deduplication and anonymisation required for populating the relational database in a platform agnostic way; and linkage and extraction; therefore it was efficient to build upon this platform to handle imaging data as well (creating the '**i**maging RDMP' or **iRDMP**).

Choice of Micro Services

The modular services/messaging/plugin architecture allows independence over the choice of underlying tools. This means that any one of the components or tools employed within the architecture can be reasonably easily swapped out for another solution and simplifies development, testing and refinement of components in isolation, minimising and containing the side-effects of changes. A microservice architecture is one which decomposes a monolithic application into a set of smaller loosely-coupled services communicating over well-defined interfaces [16]. Advantages of a Microservice architecture as opposed to a monolithic approach have been known in the IT industry in recent years (e.g. Amazon [17], Netflix [18]) and recently for health data [19].

Messaging

For the communication mechanism between the microservices, Rabbit MQ was selected as it is the most widely deployed open source message broker [14], with a robust history, active development and support community and good performance making it suitable for this project.

Non-structured database solution for identifiable metadata

The data in the DICOM tags is largely unstructured and deeply hierarchical. The flexible schema would be challenging to represent in a relational store. Moreover, its structure may change over time (e.g. new tags). Consequently, the use of a document-oriented, flexible and dynamic data storage was deemed necessary. MongoDB [20] was selected as the NoSQL database technology because the

hierarchy of DICOM tags can be mapped directly to a JSON document, then indexed and queried efficiently. This facilitated the transfer of images across database collections and use of queries against DICOM tags to select and control the promotion of data to later stages in the process.

Within the architecture the MongoDB database provided a middle ground within the ETL data flow. It allows mappings to the relational database schemas to be quickly modified and tested, while being able to quickly reload and re-process data from MongoDB rather than the slow process of going back to DICOM files. As DICOM tags are serialised to JSON for transmission through RabbitMQ, there was little developmental overhead to use the same JSON serialisation for MongoDB documents. Mongo DB has a proven ability to scale well for hundreds of millions of documents, so the schema could be practically assumed to be stable, regardless of the number or variance of records.

There is MongoDB expertise within the team of de-identification analysts, system administrators and software developers.

Structured database solution for de-identifiable metadata

The ETL microservice supports Oracle, PostgreSQL, MySQL and Microsoft SQL Server. Of these, MySQL was chosen to host the de-identifiable metadata because eDRIS already store datasets on such a database and it is desirable that these datasets are easily linked to the de-identifiable metadata data set.

Many DICOM servers and APIs have a way of representing DICOM in a relational schema e.g. dcm4chee [21]. We have used our own (dynamic) cut down schema for several reasons:

- To present something to data analysts that has a simplicity (without requiring DICOM expertise) on the same level as the other linkable datasets hosted on the National Safe Haven.
- To optimise for linkage i.e. the ability to limit the number of table joins needed and create efficient query orientated indexes e.g. PatientId+ImageType+StudyDescription
- The ability to adjust this schema and regenerate the data as future development requires.
- The ability to add additional curated fields from external sources or transformed columns such as results from expert mark-up as ground truth data.
- The ability to store (and therefore expose) a limited set of tags (those we understand will not contain identifiable data).

Anonymisation Tools

There are many different software programs which can be used to de-identify imaging data. We tested the feasibility of 3 different widely used programs (DICOM Confidential [22], XNAT [23, 24], CTP [25]) in deciding which to adopt as part of the pipeline. A summary of each is provided in Appendix B.

For a meaningful comparison of the tools, a set of criteria were devised, and each de-identification program was examined in turn against these criteria using a rating of 1-5 (where 5 is the best). We grouped the results into 3 different categories: core functionality, user friendliness and support. Table 4 shows a summary of the scores for each category with the detailed analysis provided in supplementary material A.

In summary, DICOM Confidential was ruled out due to the quality of the documentation and the lack of first-party or community support. We found that some of the images produced by DICOM Confidential were corrupted and chose not to investigate any further as the functionality of the other two tools appeared superior.

There was little difference in the functionality of CTP and XNAT. They are both well-supported tools which could perform the required tasks. The overall score of CTP was higher than XNAT. We thought that the XNAT image "bundling" for applying rules to subsets of images would be a useful capability which CTP does not provide. The pixel level anonymisation capability appeared to be much better supported and straightforward in CTP, and this is very important for this project. For these reasons we chose CTP.

Table 4 – Score of different de-identification tools

NIFTI as a method of de-identification

NIFTI (Neuroimaging Informatics Technology Initiative) is an alternative to DICOM as a medical image storage file format. Originally created for neuroimaging, NIFTI stores image data as a single 3D image (.nii file), whereas DICOM stores a separate image file for each slice of the scan. In addition, the NIFTI format only stores pixel data and metadata related to the image itself, not any patient or study information as you would find in a DICOM image. This makes NIFTI a possible method to "anonymise" DICOM images. Not all image modalities and compression methods are supported however, and conversion tools require extensions to interpret the private tags that some image scanners write into the DICOM files to describe the pixel data. Therefore, NIFTI was not chosen.

NIFTI has become popular in some machine learning applications and is preferred over DICOM due to the ease of dealing with only 1 file representing the whole 3D scan. The images for each research project can be provided in a range of formats (including NIFTI), but conversion to NIFTI was not adopted as a method for de-identification.

Pixel data and anonymisation

Primary original CT scans were found to contain no "burned-in" text i.e. no text within the pixel data. For MRI images we integrated a text-detection tool into our extraction pipeline, running each image

through the Tesseract open-source OCR tool (originally developed by Hewlett-Packard) to detect the presence of any readable text.

Software deployed in the Safe Haven Analytical Environment

We investigated several tools to deploy into the Safe Haven for managing, viewing and manual annotation of images by research teams. We chose MicroDICOM (simple DICOM viewer) [26] as the first example to use. Over time it is expected that the number of tools available as part of the pre-installed VM will increase and that researchers will have the capability to install their own preferred software tools.

Comparisons to other existing systems

Given the different imaging platforms in active development to support research projects, we investigated alternative platforms so that we did not re-invent the wheel. In general terms, other solutions have concentrated on consented cohorts from researcher collected research images rather than much larger unconsented data from routinely collected "real world" images. The architectural solution developed by others is generally a large anonymised database (sometimes distributed) containing all the images with permissions to see, extract and run pipelines on the imaging data configured for each research group. The metadata provided is limited and relatively clean in comparison to routinely collected data. The architectural challenges and solutions are therefore very different. For example, a key functionality of the platform is the efficient and effective selection of anonymised cohorts from petabytes of noisy and heterogeneous identifiable data.

If the requirements were to store a de-identifiable, clean, homogenised copy of all of the pixel and metadata within the de-identifiable zone we could have employed one of the many excellent open source platforms for managing large volumes of imaging data such as OMERO [27, 28], XNAT [23, 24], ClearCanvas [29]. There are several reasons why we did not choose this approach and therefore did not use such platforms to manage the core data repository:

- We envisage that the methods to de-identify data will change over time as our understanding increases and technological solutions improve. It is impractical to re-create >2 petabytes of de-identified images each time our methodology improves.
- It is unnecessary to undertake the effort to validate any de-identification method on all DICOM tags when only a small fraction of these will be required by research teams. It is unknown which ones will be required upfront.
- A proportion of all the images will never be extracted/released for research projects as they will not meet the cohort requirements. De-identifying imaging data reactively, only when it is required for a specific project, removes the requirement to carry out a needless timely and

computationally expensive de-identification process on images which are never required. (Conversely, a given image may be de-identified multiple times, once for each project. This is an issue which we plan to resolve in the next stage of development. We expand on this in the discussion section).

- It is risky to test a specific de-identification tool on sample data and trust that it will therefore also be successful for variations of routinely collected data from multiple sources and vendors. The architecture was designed to reduce this risk: by default blacklisting all data until proven otherwise, in which case the metadata and/or image is then "promoted" to a white list.
- The data is currently >2 Petabytes and expected to grow at ~400TB per year. There is significant cost of maintaining two copies of the data both in terms of hardware and the maintenance required to update a duplicate as new data arrives (an identifiable version of the data is required in the identifiable zone to meet requirement I – see Appendix A) .
- Hosting duplicate versions of the data introduces additional data security and governance risks.
- Different research projects require different de-identification. For example, the granularity of date and patient age data may change depending on the specific questions posed by a research project – the overarching rule is that the data be de-identified as far as possible while meeting the research requirement.
- Following the data protection principle that individuals should see the minimum data to fulfil their job role, there is no need for Research Co-ordinators to see the pixel level data to build cohorts therefore, only text-based metadata is provided for cohort building.

Although existing solutions will not fully meet the requirements of this programme, one of our core principles is to reuse as many applicable, open source or freely available tools as possible i.e. do not try to re-invent the wheel. Therefore, where relevant we have included other software within our architecture.

Testing

The SMI microservices, and the RDMP Framework upon which it relies, have been developed entirely using a Test-Driven Development approach. Continuous Integration (CI) unit and system integration tests ensure code stability. Approximately 1450 automated tests cover the core RDMP code base, and in excess of 300 tests run on the SMI microservices.

Following development of a baseline version, functional and non-functional manual testing was undertaken. The test cases were planned and documented in advance, following a series of interviews with clinicians, academics and technical staff. While these scenarios were planned, documented and

agreed, the approach to executing the tests was deliberately as exploratory as possible, rather than restricted by specific test scripting.

Exemplar-driven use cases

A number of use case scenarios were defined with input from Researchers (listing in Appendix D). These scenarios were further elaborated by a team from eDRIS, in effect, performing a dry run as though these were real research projects. The test cases assessed included:

- Cohorts can be generated using the metadata repository information
- Images can be returned where the cohort has been generated from another dataset
- Cohorts and images can be identified using a combination of the metadata repository and other data sources

Scalability Testing

Performance was benchmarked at the main processing stages of the end – to – end solution: initial load, population of the relational DB, and extraction/anonymisation.

Initial Data Load: DICOM files to Mongo DB

- Speed of population (10 reader processes running over NFS)
    - Per month: 33 hours = 5.5 days
    - Per year: 66 days
- Disk size consumed:
    - Per image: 7.2KB
    - Per month: 170GB (without indexes on series (extra 5%))
    - Per year: 2TB (without indexes on series)

Populating the Metadata Repository

- Speed of load
    - approximately 36,000 image rows per min
    - Per month: 11 hours
    - Per year: 6 days
- Disk size consumed:

- Per month: 15 GB (+ 5% metadata/archive/auditing)
- Per year: 180 GB

Extraction and Anonymisation

Test were run on increasing numbers of images, and the processing time was logged as shown in Table 5. These tests were run using CT scans only (other modalities can have significantly higher number of images per series and/or larger file sizes). Each DICOM file is around 0.5Mb. On average each CT series has 325 images giving a total file size of around 170Mb. The hardware on which this runs is summarised in Appendix C.

Table 5: Scalability of anonymisation processing

Discussion

The system we have developed is not a new tool for managing and viewing images like XNAT, OMERO, MicroDICOM and ClearCanvas. iRDMP is a platform and pipeline for extracting images from a directory of images based upon cohort selection criteria, anonymising them and copying the images into a secure location for analysis. Theoretically a tool/system for managing and viewing images from a single data store could have been configured/enhanced with a permissions layer to restrict access to only the images each research group had the right to see. This model was discounted as it did not meet the requirements for several reasons:

- Risk of hacking
- Risk of de-identification going wrong
- Speed of access
- Researchers wishing to use their favourite tools to manage and manipulate imaging data.
- Cohort building functionality and linking to other dataset is not available within such tools.
- Speed of de-identification on the fly, or needing 2 copies of all of the data.

Applicability/potential of the architecture and platform to be utilised in other environments/use cases:

There are many different platforms in active development to support multiple research projects using clinical imaging data. Our architecture has not just been designed to fulfil Scottish data governance principles and data structures, there is a much wider applicability. There are many other Safe Havens nationally and internationally [30, 31] where such a solution might be applicable and there is a trend towards the creation of new Safe Havens. Although within our architecture data extracts are viewed within a Safe Haven Analytical Platform (as part of the Scottish Data Governance requirements), the software platform can extract data to any destination. The software could therefore be utilised by

other groups/organisations to manage imaging data and build cohorts for extraction which do not use Safe Haven environments.

We have tested our software on 2 different environments with different hardware and VM tools: a regional Safe Haven and the National Safe Haven. It proved flexible enough to work in both environments.

We have created a docker based integration repository [32] which supports automated testing (in Travis CI) of the full stack of microservices with test data generated by BadMedicine.Dicom [33]. This ensures that deployment of the tech stack is simple and reproducible.

Potential impact of enabling this resource

The SMI data, linked to other datasets, along with the secure iRDMP platform we have developed has the potential to reduce the costs and widen access to large quantities of routinely collected de-identified images at scale. It also has the potential to reduce the effort of obtaining governance approval, as a Data Controller approved method for de-identification and access has already been agreed. Increasing the availability of large scale routinely collected imaging datasets linked to other forms of health data for both industry and academic use will hopefully lead to a greater likelihood of achieving results translatable into diagnoses and treatments.

Future Plans

Short term:

There are several developments which will enhance the functionality beyond that already provided which we aim to implement in the near future. Rather than the limited subset of CT and MRI metadata tags currently promoted, we plan on promoting many more of these tags. We would like to trial the use of the wider RDMP tool for cohort building and audit within the de-identifiable zone. This will require training on the tool and slight modifications to existing workflows. We would also like to fully automate the processes once the testing of the components has been completed. We are in the process of loading all historical data into the system, after which time we would like to carry out some performance testing of the solution to identify and investigate bottlenecks.

Medium term:

As well as enabling other modalities (in addition to CT and MRI), we would like to support complex cohort building:

- **Structured Reports** are summary information mainly stored in free text format which have been populated by a clinician about the study. They can include patient information such as why the

scan was requested in the first place, the condition found and family history. A cohort derived from structured reports might seek to extract all the images where a CT scan was performed because a lung tumour was expected. Structured reports are challenging to query because they can be highly identifiable, are free text and sparsely populated. As such, Natural Language processing methods have been widely utilised to extract information from the reports. We plan on utilising and extending many of these methods within the platform to extract relevant metadata from the reports which can then be utilised for complex cohort building.

- **Pixel Data** contains information which could be helpful for building a cohort of relevant images e.g. extract all x-ray images of the knee where the depth of cartilage is less than 2mm. This information is not captured in the DICOM metadata and instead would be obtained using an image processing algorithm to extract supporting features. We plan on developing automation processes where potentially relevant images are opened and the algorithm applied to the pixel data returning the cartilage depth. The cartilage depth can then be used to link with other data.
- **Complex DICOM Metadata:** The same information can be found within different DICOM tags depending on the source e.g. identifying an image as a Susceptibility Weighted Imaging (SWI) sequence requires checking three different fields for the occurrence of one of four possible strings and then filtering out some specific mismatches. This leads to problems of standardisation, metadata and definition of data dictionaries. In Scotland there are 4 different Radiology Information Systems which hold data in different ways. There are additional complexities due to conflicting requirements for standardisation for the purposes of cohort building and research use. We plan to develop algorithms for text mining and imaging metadata standardisation to provide summary data (data dimensioning) which can then be logically queried for cohort selection. We plan on investigating unsupervised machine learning techniques to group images into commonly used clusters such as body area.

Long term:

Simply copying pixel data for each research project may not scale for imaging data, where storage could quickly become infeasible as the SH hosts ever greater numbers of studies each requiring large imaging datasets. An efficient method of sharing the pixel data between multiple studies may be required. However, each study will have different metadata, e.g. study-specific patient identifiers in the image header, so a solution which combines shared pixel data with study-specific non-pixel metadata is needed. We plan to investigate different solutions such as a Virtual File Server (already developed in prototype), requiring each research group to purchase more disk space should their project require it, pulling images in batches/caching or another technical solution entirely. Different

strategies for serving images may be required, such as a file share for machine learning consumption but a DICOM server when using a DICOM image viewer.

We are fortunate to have received significant funding from the MRC and EPSRC to deliver all these future plans within a 5-year programme grant called PICTURES (InterdisciPlInary Collaboration for efficienT and effective Use of clinical images in big data health care RESearch).

We are very interested in collaborating with other groups working on any of these issues.

Limitations of the architecture

We are aware of some limitations of the current architecture:

- Huge number of images can make cohort creation cumbersome at the image level. To address this, we are adding support to the infrastructure and relational database to enable research coordinators to mostly operate at the study level.
- By not anonymising each image once on initial receipt we introduce additional complexity and increased storage requirements. In addition, we have to repeat the anonymisation task each time the image is used so we have more work to do. We trade this off against the ability to apply better anonymisation later.
- The relational database structure is not ideal for some more complex parts of DICOM. We believe that for cohort generation our flattened relational structure is simple and functional, but we may discover cases in the future where it becomes cumbersome for some parts of DICOM.

Conclusions

We have designed an architecture which meets the requirements of data governance and security and initial indications suggest that it will manage and provide extracts of routinely collected imaging data linked to other relevant datasets for research from the >2 petabytes of SMI data. We have tested the extraction system on 5 use cases, based on real exemplar scenarios.

The introduction section of this paper identified 5 challenges to using routinely collected clinical images for research. To address challenge 1, the platform uses a Data Controller approved, standardised de-identification workflow. To mitigate the risks to the Data Controllers of providing unconsented routinely collected images for research (thus addressing challenge 2) the platform works in accordance with the Scottish Government guiding principles for secure linking, anonymising and analysing data sets for research, where a subset of data for a specific cohort are linked for an approved research project and access provided via a Safe Haven (**SH**) environment (the NHS Scotland term for a

Trusted Research Environment)[34]. Access to the data can be revoked by the Data Controller at any time and researchers cannot extract/output any information other than aggregate level results from the environment. There is a separation of the roles of indexer, linker (carried out by a trusted third party or Safe Haven staff) and researcher. The platform stores the SMI data in the standard DICOM format (rather than a proprietary format) and provides the capability to search for and build research cohorts, reducing the cost of providing images for research projects and also providing a mechanism to return relevant images from different data sources (addressing challenges 4 and 5). The identifiable data is maintained to enable linkage to other datasets (addressing challenge 3) but is not released for cohort building or for research.

If you would like to access the SMI dataset for a research project, please contact eDRIS [13] in the first instance.

Availability of source code and requirements

Project name: Research Data Management Platform

Project home page: https://github.com/HicServices/RDMP

Synthetic data generation (used in integration testing):

https://github.com/HicServices/BadMedicine (EHR)

https://github.com/HicServices/BadMedicine.Dicom (DICOM data)

Template building and relational database loading library:

https://github.com/HicServices/DicomTypeTranslation (translation between DICOM types and database types, also includes example modality templates)

https://github.com/HicServices/DicomTemplateBuilder (GUI editor for building templates)

Imaging Repos

https://github.com/HicServices/RdmpDicom (RDMP plugin for imaging)

https://github.com/SMI/SmiServices (Imaging microservices)

https://github.com/SMI/Integration (Full stack testing for microservices)

Operating system(s): Windows (GUI) or Linux (console)

Programming language: C#

Other requirements: Microsoft SQL Server

License: GPL v3

No restrictions to use by non-academics.

Declarations

List of abbreviations

DB: Database

ETL: Extract Transform Load

eDRIS: electronic Data Research and Innovation Service

NHS: National Health Service

HIC: Health Informatics Centre

ISD: Information Services Division

RDMP: Research Data Management Platform

SMI: Scottish Medical Imaging

SQL: Structured Query Language

VM: Virtual Machine

Competing interests

The author(s) declare that they have no competing interests.

Funding

The authors acknowledge the support from the Farr Institute of Health Informatics Research and Dundee University Medical School. This work was supported by the Medical Research Council (MRC) grant number MR/M501633/1 (PI: Andrew Morris) and the Wellcome Trust grant number WT086113 through the Scottish Health Informatics Programme (SHIP) (PI: Andrew Morris). SHIP is a collaboration between the Universities of Aberdeen, Dundee, Edinburgh, Glasgow and St Andrews, and the Information Services Division of NHS Scotland. This project has also been supported by MRC and EPSRC [grant number MR/S010351/1] and by The Chief Scientist Office of the Scottish Government Health and Social Care Directorates via a leverage grant made to Farr Scotland. The project was also supported by the Scottish Government through the "Imaging AI" grant award.

This work was supported by Health Data Research UK which receives its funding from HDR UK Ltd (HDR-5012) funded by the UK Medical Research Council, Engineering and Physical Sciences Research Council, Economic and Social Research Council, Department of Health and Social Care (England), Chief Scientist Office of the Scottish Government Health and Social Care Directorates, Health and Social Care

Research and Development Division (Welsh Government), Public Health Agency (Northern Ireland), British Heart Foundation (BHF) and the Wellcome Trust

Authors' Contributions

EJ conceived, lead and directed the project and drafted this manuscript. GM, TN and AH designed the architecture. TN, LT, GM, PG, AH, RM, JS and RT all developed and tested the software. CS, CM, JC, DK and SP all provided requirements for the system, supported the testing of the software and provided data governance support and expertise. DS-M and JRH provide database development and support. DS, WK and DM provided operational support and development for the underpinning National Safe Haven infrastructure. AM and CS chaired and provided expertise as part of the project oversight group. AM provided leadership for this project as part of the wider Farr and HDR UK initiatives. RB and DH were project managers. All authors read, edited and approved the final manuscript.

Statement on Competing Interests

The authors have no competing interests to declare.

References

1. Snyder PJ, Johnson LN, Lim YY, Santos CY, Alber J, Maruff P, et al. Nonvascular retinal imaging markers of preclinical Alzheimer's disease. Alzheimer's & Dementia: Diagnosis, Assessment & Disease Monitoring. 4:169-78. doi:10.1016/j.dadm.2016.09.001.
2. Kickingereder P, Neuberger U, Bonekamp D, Piechotta PL, Götz M, Wick A, et al. Radiomic subtyping improves disease stratification beyond key molecular, clinical, and standard imaging characteristics in patients with glioblastoma. Neuro-Oncology. 2017:nox188-nox. doi:10.1093/neuonc/nox188.
3. Chaddad A, Desrosiers C, Toews M and Abdulkarim B. Predicting survival time of lung cancer patients using radiomic analysis. Oncotarget. 2017;8 61 doi:10.18632/oncotarget.22251.
4. Rios Velazquez E, Parmar C, Liu Y, Coroller TP, Cruz G, Stringfield O, et al. Somatic Mutations Drive Distinct Imaging Phenotypes in Lung Cancer. Cancer research. 2017;77 14:3922.
5. McGarry SD, Hurrell SL, Kaczmarowski AL, Cochran EJ, Connelly J, Rand SD, et al. Magnetic Resonance Imaging-Based Radiomic Profiles Predict Patient Prognosis in Newly Diagnosed Glioblastoma Before Therapy. Tomography : a journal for imaging research. 2016;2 3:223-8. doi:10.18383/j.tom.2016.00250.
6. Yu W, Tang C, Hobbs BP, Li X, Koay EJ, Wistuba II, et al. Development and Validation of a Predictive Radiomics Model for Clinical Outcomes in Stage I Non-small Cell Lung Cancer. International Journal of Radiation Oncology • Biology • Physics. doi:10.1016/j.ijrobp.2017.10.046.
7. Shen C, Liu Z, Guan M, Song J, Lian Y, Wang S, et al. 2D and 3D CT Radiomics Features Prognostic Performance Comparison in Non-Small Cell Lung Cancer. Translational Oncology. 10 6:886-94. doi:10.1016/j.tranon.2017.08.007.
8. Gillies RJ, Kinahan PE and Hricak H. Radiomics: Images Are More than Pictures, They Are Data. Radiology. 2015;278 2:563-77. doi:10.1148/radiol.2015151169.
9. Parmar C, Grossmann P, Bussink J, Lambin P and Aerts HJWL. Machine Learning methods for Quantitative Radiomic Biomarkers. Scientific reports. 2015;5:13087. doi:10.1038/srep13087

https://www.nature.com/articles/srep13087#supplementary-information.
10. Hsu C-Y, Doubrovin M, Hua C-H, Mohammed O, Shulkin BL, Kaste S, et al. Radiomics Features Differentiate Between Normal and Tumoral High-Fdg Uptake. Scientific reports. 2018;8 1:3913. doi:10.1038/s41598-018-22319-4.
11. Lambin P, Leijenaar RTH, Deist TM, Peerlings J, de Jong EEC, van Timmeren J, et al. Radiomics: the bridge between medical imaging and personalized medicine. Nature Reviews Clinical Oncology. 2017;14:749. doi:10.1038/nrclinonc.2017.141

https://www.nature.com/articles/nrclinonc.2017.141#supplementary-information.
12. DICOM Standard. https://www.dicomstandard.org/current/.
13. electronic Data Research and Innovation Service (eDRIS). http://www.isdscotland.org/Products-and-Services/eDRIS/.
14. RabbitMQ: RabbitMQ is the most widely deployed open source message broker. https://www.rabbitmq.com/.
15. Nind T, Galloway J, McAllister G, Scobbie D, Bonney W, Hall C, et al. The Research Data Management Platform (RDMP): A novel, process driven, open-source tool for the management of longitudinal cohorts of clinical data. GigaScience. 2018:giy060-giy. doi:10.1093/gigascience/giy060.
16. Martin Fowler: Microservices. https://martinfowler.com/articles/microservices.html.
17. Amazon: What are Microservices? https://aws.amazon.com/microservices/.
18. Adopting Microservices at Netflix: Lessons for Architectural Design. https://www.nginx.com/blog/microservices-at-netflix-architectural-best-practices/ (2015).

19. Yang Y, Zu Q, Liu P, Ouyang D and Li X. MicroShare: Privacy-Preserved Medical Resource Sharing through MicroService Architecture. International journal of biological sciences. 2018;14 8:907-19. doi:10.7150/ijbs.24617.
20. MongoDB: Flexible enough to fit any industry. https://www.mongodb.com/who-uses-mongodb.
21. dcm4chee: Database Table Descriptions. https://dcm4che.atlassian.net/wiki/spaces/ee2/pages/2556012/Database+Table+Descriptions.
22. Rodríguez D, Carpenter TK, van Hemert JI and Wardlaw JM. An open source toolkit for medical imaging de-identification. European Radiology. 2010;20 8.
23. Abbott D: What is Digital Curation? http://www.dcc.ac.uk/resources/briefing-papers/introduction-curation/what-digital-curation (2014).
24. Marcus DS, Fotenos AF, Csernansky JG, Morris JC and Buckner RL. Open Access Series of Imaging Studies: Longitudinal MRI Data in Nondemented and Demented Older Adults. Journal of Cognitive Neuroscience. 2010;22 12:2677-84. doi:10.1162/jocn.2009.21407.
25. Aryanto KYE, Broekema A, Oudkerk M and van Ooijen PMA. Implementation of an anonymisation tool for clinical trials using a clinical trial processor integrated with an existing trial patient data information system. European Radiology. 2012;22 1:144-51. doi:10.1007/s00330-011-2235-y.
26. MicroDicom. http://www.microdicom.com/.
27. Swedlow JR. Informatics and Quantitative Analysis in Biological Imaging. Science. 2003;300 5616:100-2. doi:10.1126/science.1082602.
28. Williams E, Moore J, Li SW, Rustici G, Tarkowska A, Chessel A, et al. The Image Data Resource: A Scalable Platform for Biological Image Data Access, Integration, and Dissemination. Cold Spring Harbor Laboratory, 2016.
29. Synaptive Medical: ClearCanvas. https://www.clearcanvas.ca/.
30. Lea NC, Nicholls J, Dobbs C, Sethi N, Cunningham J, Ainsworth J, et al. Data Safe Havens and Trust: Toward a Common Understanding of Trusted Research Platforms for Governing Secure and Ethical Health Research. JMIR Medical Informatics. 2016;4 2:e22. doi:10.2196/medinform.5571.
31. Burton PR, Murtagh MJ, Boyd A, Williams JB, Dove ES, Wallace SE, et al. Data Safe Havens in health research and healthcare. Bioinformatics. 2015;31 20:3241-8. doi:10.1093/bioinformatics/btv279.
32. Integration Code. https://github.com/SMI/Integration.
33. BadMedicine DICOM code. https://github.com/HicServices/BadMedicine.Dicom.
34. SHIP Guiding Principles and Best Practices. http://www.scot-ship.ac.uk/sites/default/files/Reports/Guiding_Principles_and_Best_Practices_221010.pdf (2010).

 Click here to download Table Tables.pdf

Data	Description
DICOM Files (unmodified historic data)	DICOM files are stored unaltered in a file archive. There are many reasons why we wish to keep the identifiable data and store the original DICOM images:<br>• Any program developed to strip all identifiable data from the DICOM files and tags risks rendering the whole dataset unusable if done incorrectly. Linkage to other datasets would subsequently be either incorrect or impossible.<br>• It is conceivable that future data de-identification strategies will wish to make use of some identifiable data and removing that data would therefore limit future options.<br>• The NHS may wish to use the data as a secondary offline Disaster Recovery system or use the data to populate a clinical system from an alternative provider. In which case it needs to be technically feasible to generate the data in identifiable form in a format that is non-proprietary and as close as possible to the DICOM files as they were originally captured.
Identifiable DICOM Tag Data	All tag metadata from the DICOM files are extracted to a MongoDB database in a searchable format.<br>This tag metadata is stored in an identifiable format because De-identification Analysts need to know what the identifiable data is so that they can remove it *e.g.*<br>• If the patient name is Mrs Jones then if searching for identifiable data in the clinical report the De-identification Analyst will need to know to look for the text "Jones" in order to remove this data.<br>• Or if checking if an image is identifiable, they might need to know the CHI number in order to check this is not burnt into the pixel data.
Inventory Tables	A subset of data from the Identifiable set above is copied here.<br>This is a relational database which contains suitably cleansed and de-identified image metadata (and file paths), i.e. has been confirmed to be well-populated, of high quality and does not contain identifiable data. This is used by the Research Co-ordinators for cohort creation and extraction to the Safe Haven. The data is indexed using EUPIs.<br>For example, DICOM age strings can express the age in years, months or days (e.g. 075Y, 006M or 002D). The cleaned and homogenised metadata will store these in a consistent and easily queried numeric format.

	Other metadata fields may be a single value summarising data stored in multiple different DICOM tags. For example, by analysing the acquisition position of the images it is possible to identify examinations where the same volume has been acquired repeatedly in a single series, when used in conjunction with tags to show if contrast was used during the examination this can be used to disambiguate contrast bolus imaging from other acquisitions that may also use contrast.
Cohort and associated Anonymous Research Extracts	Any research project will start by defining a relevant cohort and obtaining the necessary ethical/administrative approval in consultation between the Researchers and the Research Coordinators – this is an out of band process outwith the iRDMP system, so not shown here.<br><br>The Data Analysts then assemble a data set (the Anonymous Research Extract) for that research project by querying the Inventory Table, possibly linked against other data sources via the EUPI (pseudonymised patient ID, explained below) and trigger the Extraction Microservices to export the appropriate subset of columns made available to the research users. For example, a project might request all available brain MRI scans from patients who have been prescribed Gabapentin, and want the dosage information and patient age; they would be given a set of image data (the scans themselves, passed through the DICOM file anonymiser described later) and a table of associated metadata including the dosage information for each de-identified patient.
Research Data Management Platform (RDMP)	The RDMP manages and monitors the extraction processes.
CHI to EUPI Mapping Table	Scotland uses the CHI unique identifier for health data. Adhering to the Guiding Principles of data linkage for research [14], the National Safe Haven separates out the roles of indexer and linker. Research Co-ordinators link data from a range of sources provided to them with the CHI replaced by the EUPI identifier. The imaging data also follows this methodology. The mapping table is securely held and is only accessible via the automated conversion process. An individual can be given multiple CHIs if they access healthcare in different regions and it takes time to resolve, therefore the mapping table is updated monthly.

Table 1 - Overview of Data stores

Processes	Description
Promotion of de-identifiable tags/metadata	Promotion is a two-stage process. The first stage promotes images for which there is an anonymisation protocol (e.g. CT images). Anonymised tag data is pushed to the Inventory Table. These tables support the extraction processes and support routine practices e.g. data cleaning. A subset of this data (e.g. only PRIMARY/ORIGINAL images) is pushed to the De-Identified Zone indicating the images can be used for cohort generation / image extraction. This data push can include collapsing data e.g. to Series/Study level.<br><br>It is not feasible or desirable to proactively analyse the complete Identifiable DICOM Tag Data in order to promote all tags. This is in part due to the difficulty in determining that a tag of a certain type does contain identifiable information for a) the whole of the current archive and b) future PACS images that will be taken.<br><br>A tag can be promoted in two circumstances:<br><br>1) it is determined not to contain identifiable information,<br><br>2) or the identifiable information it does contain can be de-identified.<br><br>Sophisticated techniques such as Natural Language Processing methodologies can be used to determine condition 1 or find a solution for de-identification for condition 2. The solution for condition 2 is known as an anonymisation profile and can be saved for reuse. Once a tag can be flagged as safe for promotion it is moved to the Inventory Table.<br><br>This is an iterative process (future studies with unique requirements will inform which data is prioritised for anonymisation/promoted).
Promotion of image types which are extractable	This process white-lists images which are extractable in the sense that pixel data can completely be de-identified. Some images, particularly ultrasounds, may have identifiable information such as patient name or CHI watermarked on the image. Which images can be de-identified is stored in the metadata catalogue but the rules regarding how images are de-identified are stored in CTP [17] anonymisation scripts that apply to all images. These scripts contain rules such as "if Modality is US and Manufacturer is X and model is Y then blank out pixels in the rectangle (0,0,1000,200)".
Mapping (CHI-EUPI)	This process is called when metadata is promoted to the de-identifiable zone to replace identifiable CHIs with EUPI. It is an automated process so that no individuals can see this mapping.

Cohort Creation Process	A set of software tools (or manual SQL queries if the user prefers) which query the DICOM metadata within the Inventory Tables in order to select images relevant for a particular cohort (by applying filters which describe researcher requirements). The resulting cohort forms the basis for both the initial and subsequent releases of data to the Safe Haven for the relevant study, and as such it is critically important that the cohort is identified and managed correctly.
Extraction Process	This process uses the Cohort database and Inventory Tables to determine which files to extract for a particular research project. It calls the DICOM file anonymiser to de-identify the relevant files used to build the cohort for release to the researcher. After the cohort output and the de-identified DICOM files are curated, the process triggers a release into the Researcher Safe Haven environment.
DICOM file anonymiser	The DICOM file anonymiser:<br>• Obtains the file(s) from the file archives<br>• Anonymises the pixel data of the file if necessary<br>• Anonymises the metadata in the file (leaving only the whitelisted tags)<br>• Converts the file to an alternative format if required<br>• Returns the final file(s) to the user
Researcher VM with tools to view and manipulate images	There are 2 main use cases: small scale studies where a researcher team may wish to open and mark up each image by eye and large scale studies where software and algorithms will be developed by the users of the system to analyse the images for their specific project. The different tools available within the Safe Haven meet both sets of requirements. The researcher VM image includes a standard set of tools which will be increased over time as the requirements increase. Example tools are MicroDICOM (simple DICOM viewer), ClearCanvas (open-source PACS client, cf. Carestream) and XNAT. The VM should have the capability for users to securely add their own tools. The VM provides access to the associated data from study-specific image metadata and pixel data but does not allow row level or pixel data to be extracted. Access to the internet is restricted when analysing the data.

Table 2 - Overview of Processes

Role	Description
Researchers	Carry out the research on a dataset extracted from the SMI DB and other linked data. Any project may have a variety of researchers including clinicians, statisticians, radiographers, image analysis and machine learning experts etc. They view and work on the PACS images within a Safe Haven environment.
Research coordinators/cohort builders	Work with the Researchers to produce the data extract that allows the research study to be carried out. Research coordinators understand where the data is stored, how to link across datasets and will run software, write scripts, query databases etc. in order to produce the final cohort datasets.
Data analysts	Work with de-identified PACS data to produce more usable versions of the data for research coordinators to work with. Over time data analysts (working with domain experts) may produce additional mapping tables and categorisation systems that make it easier for Researchers and Research Co-ordinators to work with the data.
De-identification analysts	Are responsible for ensuring as much data as possible is made available to research coordinators for the creation of cohorts but that no identifiable data reaches the coordinators. Much of the de-identification task is automated but the system needs to be continually monitored and new DICOM tags added to the whitelist (or blacklist) as required.
System Administrators	Are part of the infrastructure team and are responsible for building and maintaining the underpinning infrastructure, security, network separation, monitoring and supporting automated processes. Supported automated processes would involve checking for example, whether there were errors in the data load process or data extraction process. They have privileges and expertise to debug and/or restart these processes.
Software developers	Produce any new software required within any zones of the environment. The software is developed and tested outside of the production environment. Deployment of software updates will be carried out by System Administrators.

Table 3: Roles

	**Core Functionality (max possible = 45)**	**User friendliness (max possible = 30)**	**Support (max possible = 25)**	**Total**
**XNAT**	37	21	22	80
**CTP**	41	24	25	90
**DICOM Confidential**	35	24	14	73

Table 4 – Score of different de-identification tools

Number image ids	Anonymisation run time	Total file size	Average run time per file
3	Negligible	1.5Mb	Negligible
2264	0.3 hours	1.2Gb	0.54 secs
89415	1.5 hours	50Gb	0.06 secs
~1.2 million	28 hours	630Gb	0.06 secs
~17.2 million	65 hours	8.5 Tb	0.01secs

Table 5: Scalability of anonymisation processing

Figure
Click here to download Figure Figure 1 - iRDMP Architecture.pdf

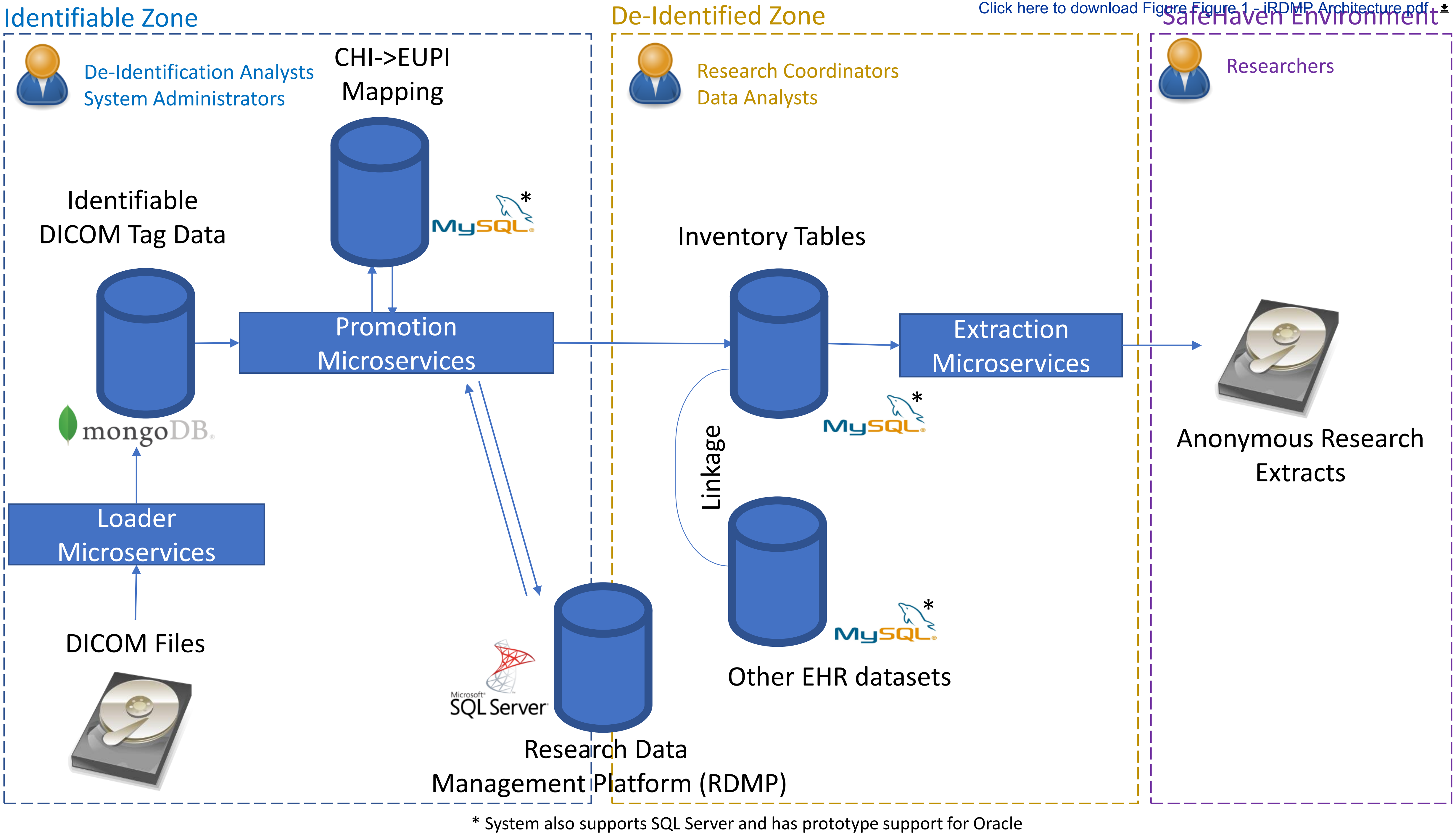

Click here to access/download
Supplementary Material
iRDMP Supplementary Material.pdf

Personal Cover Click here to download Personal Cover iRDMP Cover Letter - GigaScience.pdf

To

Laurie Goodman, PhD
Editor-in-Chief
GigaScience – Oxford Academic

March 11, 2020

Dear Dr Goodman

We are pleased to submit an original technical note entitled "An extensible big data software architecture managing a research resource of real-world clinical radiology data linked to other health data from the whole Scottish Population" for consideration for publication in GigaScience.

In 2018 we published our related paper "The research data management platform (RDMP): A novel, process driven, open-source tool for the management of longitudinal cohorts of clinical data" in gigascience (https://doi.org/10.1093/gigascience/giy060). This paper represents a major enhancement to the RDMP to enable it to support over 2.5 Petabytes of Clinical Imaging data collected since 2006 covering the whole Scottish Population. The software is available opensource for other groups to utilse and build upon.

We believe that this article is best suited for publication by GigaScience because it aligns with our previous work published by yourselves, the high impact of gigascience, and because we are strongly supportive of GigaScience's ethos of open access and reproducible research.

This research has not been published and is not under consideration for publication elsewhere. The authors have no conflicts of interest to disclose and approved the article for submission to GigaSscience.

Thank you for your consideration!

Sincerely

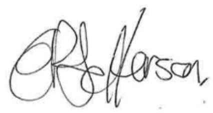

Emily Jefferson, PhD
Professor of Health Data Science and Director of the Health Informatics Centre (HIC)
University of Dundee
Dundee
DD1 9SY

erjefferson@dundee.ac.uk
+44 1382 383943
