# Supplementary material for: An extensible big data software architecture managing a research resource of real-world clinical radiology data linked to other health data from the whole Scottish population
Source: Gigascience. 2020 Sep 29;9(10):giaa095. doi: 10.1093/gigascience/giaa095 (PMC7523405; doi:10.1093/gigascience/giaa095)
Supplement: giaa095_Supplemental_File [file giaa095_supplemental_file.docx]

# Appendix A: Summary of requirements

**Main Requirement:**

1. To provide a secure method for hosting >2 Petabytes of identifiable imaging data and provision de-identified subsets of this data, linked to other datasets, for specific cohorts within a virtual Safe Haven Environment for researchers to analyse but not remove the data.

**Data Governance Requirements:**

1. To adhere to the Scottish Safe Haven Charter [35] for the use of unconsented data for research and work within the existing National Safe Haven architecture.
2. To satisfy data governance requirements so that there is clear separation of roles for the users of the platform and only the minimum amount of data can be seen to fulfil each role. Therefore:
   - Researchers can only see a de-identified subset of data which is required to answer their specific research question.
   - Researchers cannot build cohorts directly themselves from the raw underpinning data.
   - The eDRIS team of Research Co-ordinators and Data Analysts (termed Research Co-coordinators throughout this document) who provision data extracts for the researchers can only see a de-identified version of the metadata about the images in order to fulfil the role of cohort building.
   - Only individuals who are maintaining the infrastructure or fulfilling the role of de-identification analyst can view identifiable data and only when carrying out specific tasks that require them to view identifiable data.
3. To protect identifiable data from unauthorised access.

**Cohort Building Requirements:**

1. To support the National Safe Haven Research Co-ordinators to build cohorts based on data from different sources such as:

- Image metadata (DICOM tag data), e.g. select MRI images of the head
- Image pixel data, e.g. select lung scans images where the airways are less than 3mm using an algorithm which extracts features from pixel data.
- Other health data sets not held in the image store (such as prescribing data), e.g. select all images where a patient has been given a particular drug within 3 years prior to the scan date.
- Other non-health related data, e.g. select images where the patient lived in a care home at the time of the scan.
- Structured reports, e.g. select images where the diagnosis of condition is the heart is enlarged or where the lungs are clear.
- Metadata about an image captured as part of the research output of a project which used the environment. There are several instances where a research project which uses the environment may curate or add value to images through their expertise. An example might be where a project requires chest CT scans and funds a radiologist to view the images and generate a gold standard curated set of 1000 images by recording whether or not the image shows evidence of coronary artery calcium. If the research group who creates the gold standard data wishes, the system should be able to record such information and use it to build further cohorts for other research projects e.g. select images where a radiologist has recorded the image as showing signs of coronary artery calcium and controls.

1. To provide technological solutions which the Research Co-ordinators are familiar with so that the same skill set can be employed e.g. use of structured SQL databases rather than un-structured databases and no software programming expertise.
2. To provision summary, curated data (feature extraction) for cohort building rather than requiring the Research Co-ordinators to require domain knowledge of the DICOM standard and the intricacies of the alternative use of the standard by different vendors, health boards and users.
3. To provide the data to the Research Co-ordinators in a form which is easily linkable to other datasets for efficient cohort building.

**Data Requirements:**

1. To preserve original data in the state it is provided. This requirement must be balanced against the data security and information governance requirements which arise from holding unconsented patient data. The goal of only discarding operationally non-critical data helps ensure that nothing is thrown away which later turns out to be important, as it will be very difficult (if not impossible) and very costly to re-download missing data from the source system. This is also important for reproducibility.
2. To minimise the number of copies of the data where possible. This is both for data governance reasons and because of the large volumes of data i.e. cost of storage and challenge of maintenance.

**System consistency:**

1. To ensure all procedures are traceable and reproducible through auditing and atomicity of operations. For example, information about the what, how or when a system user interacted with the system is stored and the interaction can be replicated.
2. To maximise data integrity by ensuring no operation whatsoever can damage production data or leave a production dataset in an indeterminate state.
3. To modularise the software components to mitigate point of failure risks, maximize reusability and dissemination of implementations.

**Efficiency and Maintainability:**

1. To reuse as many applicable, open source or freely available tools as possible i.e. do not try to re-invent the wheel.
2. To be cost effective to run and for data to be securely available for research projects in a timely fashion. A pragmatic consequence of this high level requirement is that the system should not try to de-identify all 1.5 Petabytes of data prior to the platform being utilised for research, rather it should support reactive de-identification based upon the image types required for research projects which use the system. The DIOCM standard has ~4000 different metadata tags and unspecified numbers of additional private tags. The format supports hierarchical tags (sequences) which can include a mixture of sensitive and insensitive data. The DICOM standard is used differently by different vendors, for different modalities and by different health boards resulting in highly heterogeneous data. Although there are many software programs which claim to de-identify imaging data there is a risk that such programs do not do this for all variations of the DICOM data used from such a diverse set of real-world, routinely collected imaging data. Any programme of work to develop de-identification protocols for individual machines covering the entirety of the dataset would take an infeasible amount of time. It may also be the case that a significant proportion of the imaging might never be requested for extraction, rendering unnecessary any work done to create related de-identification protocols.
3. To be vendor agnostic and open source.
4. To be able to support modular enhancements over time from other sources. There is vast expertise and other software tools which could improve the platform for the whole community. This needs to be balanced with the IG considerations and the stability of a production system.

**Research Use:**

1. To provide tools within the Safe Haven Analytical Platform which can view and manipulate images.
2. To be able to develop software within the Safe Haven Analytical Platform and a secure method for extracting the code from the environment (without being able to extract the image or image metadata).
3. For the access to the data within the Safe Haven Analytical Platform to be high performance to support deep learning and machine learning.
4. The de-identification process should not remove metadata which is required by software to view and manipulate images.
5. Different research projects may require different de-identification protocols depending on the research question they are asking. E.g. the default de-identification method would mask the exact time and date of a scan to reduce the chance of inadvertent re-identification of and individual based upon personal knowledge of when a scan was taken. However, if assessing the quality of care based on the time and day of the week the scan was taken, dates may be required.

# Appendix B: Analysis of Anonymisation Tools

## Summary of the tools

### CTP:

CTP is the short name for the Medical Imaging Resource Center (MIRC) Clinical Trials Processor[25]. It used by radiology sites participating in multisite clinical trials to manage, process and transmit the medical images and their associated information. It features processing pipelines that the imaging datasets can be passed through as preparation for their use in research, including stages for anonymisation of the DICOM data. It is written in Java.

### XNAT

XNAT is an imaging informatics platform developed by the Neuroinformatics Research Group at Washington University, USA. It is designed with extensibility and customisability in mind (usually via third party or user-defined plugins), but has a core set of functional tasks common to most uses: data upload; data organisation and sharing; data viewing and downloading; secure and managed access; searching large data sets; and running complex processing on the data. It is written in Java.

### DICOM Confidential

DICOM Confidential is a DICOM anonymisation tool first developed at the University of Edinburgh in 2010 used by some imaging centres in Edinburgh. It comes with a graphical user interface and supports a common set of anonymisation tasks and can also be extended. It is written in JAVA.

## Core functionality

The core functionality was assessed. Here we provide examples of how the operations are undertaken via the respective tools, to give a flavour of the syntax necessary. The XML syntax for DICOM Confidential is long and not easily human readable so here we simply list the name of the transformation that would be used.

### Map a tag value to a new value using an external lookup

CTP can do this straightforwardly but only for a lookup file – this may raise some issues for our database solution. XNAT can do this but requires an external app to inject the new details into the script. DICOM confidential can do this via an external file.

| Task | Map (1010,0020) Patient ID “Case Report 1” to “1234” via an external lookup |
| --- | --- |
| CTP | @lookup(ElementName, KeyType) |
| XNAT | // Example for patientID  patientID := (1010,0020) // Sets the variable patientID to the initial value  describe patientID "ChangeME" // Sets an external label for the patient ID for use by the app  (1010,0020) := patientID // DICOM attribute gets set with the new variable |
| DICOM Conf | uk.ac.ed.dcmconf.transformer.idmapper.StudyIDMapper |

### Replace a value

| Task | Replace value for (0010,0010) PatientName to “XXX” |
| --- | --- |
| CTP | PatientName = “XXX” |
| XNAT | (0010,0010) := “XXX” |
| DICOM Conf | uk.ac.ed.dcmconf.transformer.field.StringOverwriter |

### Blank a value

| Task | Blank out the value for (0008,1050) PerformingPhysicianName |
| --- | --- |
| CTP | @empty(PerformingPhysicianName) |
| XNAT | (0080,1050) := “” |
| DICOM Conf | uk.ac.ed.dcmconf.transformer.field.StringOverwriter |

### Reduce granularity of a date

No direct command for this in XNAT but easy to write one.

| Task | Change (0008,0020) StudyDate to the first of the month |
| --- | --- |
| CTP | @modifydate(this,*,*,1) // assuming **this** is set to StudyDate |
| XNAT | myStudyDate := (0008,0020)  myMonthYear := substring(myStudyDate, 4, 10)  (0008,0020) := concatenate("01/",myMonthYear) |
| DICOM Conf | uk.ac.ed.dcmconf.transformer.field.DateTransformer |

### Remove all private data

| Task | Remove all private data i.e. (7FE1,x) for all x |
| --- | --- |
| CTP | Checkbox on UI “Remove Private Groups” |
| XNAT | **-** (7FE1,XXXX) |
| DICOM Conf | There is a transformer to do this. |

### Remove a tag

| Task | Remove tag (0008,0081) InstitutionalAddress | |
| --- | --- | --- |
| CTP | @remove() | |
| XNAT | **-** (0008,0081) | |
| DICOM Conf | uk.ac.ed.dcmconf.transformer.object.AttributeRemover |  |

### Additional core functionality

Other factors considered were:

- Whitelisting tags
- Adding bespoke anonymisations
- Defining subsets of images to which the rules will apply
- The ability to anonymise pixel data

## User friendliness

The evaluation of the user friendliness of the tools was considered from two points of view:

1. The person writing and maintaining the rules
2. The system administrator installing and maintaining the software

By their nature these criteria will be more subjective.

## Support

The criteria considered included existence of an active group of developers regularly working on the software, availability of good documentation etc.

## Evaluation analysis

To give a more quantitative analysis following the evaluation, each tool was given a score between 1 (poor) and 5 (great) as to how straightforward the task was to achieve, or if there is support for the required feature and so on. Table 1,Table 2 and Table 3 show the scoring for the categories of Core Functionality, User Friendliness and Support respectively.

Table 1: Core functionality

| **Category** | **Tool** | **Comment** | **Score** |
| --- | --- | --- | --- |
| Remove a tag | CTP |  | 5 |
|  | XNAT |  | 5 |
|  | DC |  | 5 |
| Replace tag with hardwired value | CTP |  | 5 |
|  | XNAT |  | 5 |
|  | DC |  | 5 |
| Reduce granularity of a date | CTP |  | 5 |
|  | XNAT | No direct date command but do-able | 4 |
|  | DC |  | 5 |
| Map a value using database lookup | CTP | Mapping by file is default, database lookup needs bespoke extension | 4 |
|  | XNAT | Needs a bespoke plugin to do it, but it is supported | 4 |
|  |  | Mapping by file is default, database lookup need bespoke extension. | 4 |
| Remove all private data | CTP |  | 5 |
|  | XNAT |  | 5 |
|  | DC |  | 5 |
| Whitelist tags | CTP | Use the remove unchecked elements option. | 5 |
|  | XNAT | Can be done on initial import^[[1]](#footnote-1)^ but is was not clear how to do it via scripts | 3 |
|  | DC | Looks like there is a transformer to do this (untested by us) | 5 |
| Add bespoke anonymisations | CTP | Supported and well documented. In Java. | 5 |
|  | XNAT | Supported, java | 5 |
|  | DC | Supported, Java. | 5 |
| Define subsets of images to which rules apply | CTP | Supports within tag if statements that allow some degree of conditionals | 2 |
|  | XNAT | Can create "bundles"^[[2]](#footnote-2)^ which makes subsets available to others - should be possible to use this or similar to define subsets for our use | 3 |
|  | DC | Does not look possible | 2 |
| Pixel data anonymisation | CTP | Claims to do it well and have rules of all known cases | 5 |
|  | XNAT | Images can be updated^[[3]](#footnote-3)^ | 3 |
|  | DC | Claims to support this. Not been able to get working. | 3 |

Table 2: User friendliness

| **Category** | **Tool** | **Comment** | **Score** |
| --- | --- | --- | --- |
| User friendliness of rules text | CTP | XML but quite flat and readable XML | 3 |
|  | XNAT | Straightforward syntax | 4 |
|  | DC | XML too ugly for users. Refers to full classpath etc. | 2 |
| User friendliness of rule GUI | CTP | Fairly clean but doesn’t parse the operation text | 4 |
|  | XNAT | Fine | 4 |
|  | DC | Poor, clunky and does not specify what parameters are needed. | 3 |
| Ability to write new GUI as part of other tools | CTP | Would be easy to write new GUI to spit out the same XML format | 5 |
|  | XNAT | Is supported via plugin development framework | 3 |
|  | DC | Would be easy to write new GUI to spit out the same XML format. | 5 |
| Ease of use of imagined best GUI for eDRIS staff | CTP | This is one of by possible concerns. The rules are written as text, e.g. @modifydate(this,*,*,1) and @lookup(this,pid). These are fine for developers, but it would be hard to use a GUI to hide this and expose a simpler parameters and values viewpoint. | 3 |
|  | XNAT | The rules use tags to reference what to change e.g. (0008,0080) := “Hospital A” so shares issue with CTP | 3 |
|  | DC | Rules have clear parameters and default values so a well written GUI could really help the user create rules | 5 |
| Command line invocation | CTP | Points to a directory and processes all files in it | 5 |
|  | XNAT | Linux only but expected it can be adapted for other platforms given the underlying software runs anywhere | 3 |
|  | DC | Points to a directory and processes all files in it. | 5 |
| Programmatical invocation | CTP | Not formally documented but it would be fairly easy to take the command line code and repackage that | 4 |
|  | XNAT | Yes, via plugin development^[[4]](#footnote-4)^ and API ^[[5]](#footnote-5)^ | 4 |
|  | DC | Not formally documentation but it would be fairly easy to take the command line code and repackage it. | 4 |

Table 3: Support

| **Category** | **Tool** | **Comment** | **Score** |
| --- | --- | --- | --- |
| Active development | CTP |  | 5 |
|  | XNAT | Active group of developers | 5 |
|  | DC | Original developer still support it. | 3 |
| Responsive to queries | CTP | Developer made a change overnight to the command line version following a request | 5 |
|  | XNAT | No personal experience of this but the community appears to be very active | 4 |
|  | DC | Original developer responded to personal emails | 4 |
| User documentation | CTP | Good webpage explaining the anonymisation operations | 5 |
|  | XNAT | Very good, comprehensive | 5 |
|  | DC | Very poor and we had to ask the developer for it. | 2 |
| Open source | CTP | RSNA MIRC public license ^[[6]](#footnote-6)^. | 5 |
|  | XNAT | Yes – just requires the inclusion of copyright notice in redistributions | 5 |
|  | DC | Available on-line for free but just JAR files and not the latest code. Developer gave me JAR files with the code. | 3 |
| Runs without bugs or obscure limitations | CTP | No bugs or limitations discovered in initial experiments | 5 |
|  | XNAT | Currently only linux command-line. No bugs spotted. | 3 |
|  | DC | Does not run on 64 bit windows. DICOM viewer unable to view the output images. | 2 |

# Appendix C: Summary of the hardware infrastructure

The prototyping environment within the Health Informatics Centre consists of Windows 7 and 2008 R2 virtual machines under VMWare ESX with a Fibre Channel SAN.

Integration and staging are mainly done using Travis and Appveyor cloud virtual machines, running Ubuntu Linux “Bionic” (18.04 LTS) and Windows Server 2019 respectively.

The production environment built for this project within the National Safe Haven (NSH) in the Edinburgh Parallel Computing Centre has a variety of nodes connected via 10G Ethernet. The bulk storage (used mainly for holding the raw DICOM files) consists of around 3PB of space across a BeeGFS cluster, with two data nodes (each 128GB RAM, dual Xeon processors and 7 x 4TB SATA hard drives) used to hold the project data. One is configured as a RAID6 (high resilience, low write performance) array and exported to the other nodes via NFS, the other is configured as RAID10 equivalent under ZFS and hosts the MySQL database directly, after early attempts to host that on an NFS mount proved unsatisfactory. Several virtual machines access these two servers and the BeeGFS storage to run all stages of the pipeline, along with the MongoDB and RabbitMQ servers (with their data stored over NFS). All nodes run CentOS 7.3, except a monitoring machine used to configure early stages, which currently runs Windows. After resolving the write performance issues from storing MySQL database content on a RAID6-backed NFS mount, the RabbitMQ disk access became the limiting factor, but throughput is sufficient to ingest the current data set within months and then keep up with a live PACS feed once that can be provisioned.

File system metadata and image metadata will be stored on ultra-fast SSDs with an open source data management tool providing the necessary access controls and audit logs. The environment currently supports 135 data linkage research projects.

# Appendix D: Use cases

|  |  |
| --- | --- |
|  |  |
|  |  |
|  |  |
|  |  |
|  |  |

| **Images and associated linked health data from Cohort** | **Filters** |
| --- | --- |
| Lung cancer scans | CHI List defined by filtering based on SMR06, SMR01 to identify lung cancer |
| Women who gave birth between 2000 and 2005 who were a smoker before pregnancy and were 30 years or older | Demographics |
| Abd/chest CT images | Body area |
| Abdomen CTs for Colon Cancer | Cohort filtering on clinical RIS type data |
| Chest CT based on link to clinical & inflammatory data. | Complex filtering and linkage (linked to IHD as the main diagnosis from hospital inpatient episodes for calendar year 2016). |

1. <https://wiki.xnat.org/docs16/xnat-configuration-framework/series-import-filter-configuration?src=sidebar> [↑](#footnote-ref-1)
2. <https://wiki.xnat.org/docs16/3-administrator-documentation/managing-sites-projects/sharing-stored-searches> [↑](#footnote-ref-2)
3. <https://wiki.xnat.org/documentation/how-to-use-xnat/image-session-upload-methods-in-xnat/where-anonymization-happens-in-xnat> [↑](#footnote-ref-3)
4. <https://wiki.xnat.org/display/XNAT17/Creating+an+XNAT+Plugin+Project> [↑](#footnote-ref-4)
5. <https://wiki.xnat.org/documentation/the-xnat-api> [↑](#footnote-ref-5)
6. <http://mirc.rsna.org/rsnapubliclicense.pdf> [↑](#footnote-ref-6)
