# Supplementary material for: An extensible big data software architecture managing a research resource of real-world clinical radiology data linked to other health data from the whole Scottish population
Source: Gigascience. 2020 Sep 29;9(10):giaa095. doi: 10.1093/gigascience/giaa095 (PMC7523405; doi:10.1093/gigascience/giaa095)
Supplement: giaa095_Response_to_Reviewer_Comments_Original_Submission [file giaa095_response_to_reviewer_comments_original_submission.pdf]

Author's Response To Reviewer Comments

Close

We thank the reviewers for their extremely helpful comments. We have addressed all their points and attach edited new manuscripts and supplemental materials (versions with tracked changes on and off). A response to each of the reviewer's comments is provided below including where we have edited the manuscript. The reviewer comment is marked with a "*" and our response "#". We have also attached this information in a PDF document which supports additional formatting for ease of reading.

Reviewer 1
* I thank the Authors for presenting a promising solution for creating a large dataset of medical data, particularly focused on imaging. Some comments:
* The references in the opening of the Background are relatively old (e.g. ref 9 is a review from 2015), there are more recent publications that could be cited (e.g. doi: 10.1186/s41747-019-0109-2).

9 new references have been added (page 1 and 10). (these do not appear as tracked changes but have been changed):

Ahmed, L., Iqbal, M. M., Aldabbas, H., Khalid, S., Saleem, Y., & Saeed, S. (2020). Images data practices for semantic segmentation of breast cancer using deep neural network. Journal of Ambient Intelligence and Humanized Computing. doi: 10.1007/s12652-020-01680-1
Cuocolo, R., Cipullo, M. B., Stanzione, A., Ugga, L., Romeo, V., Radice, L., … Imbriaco, M. (2019). Machine learning applications in prostate cancer magnetic resonance imaging. European Radiology Experimental, 3(1), 35. doi: 10.1186/s41747-019-0109-2
Hu, Q., Whitney, H. M., & Giger, M. L. (2020). A deep learning methodology for improved breast cancer diagnosis using multiparametric MRI. Scientific Reports, 10(1), 10536. doi: 10.1038/s41598-020-67441-4
Jain, R., Jain, N., Aggarwal, A., & Hemanth, D. J. (2019). Convolutional neural network based Alzheimer's disease classification from magnetic resonance brain images. Cognitive Systems Research, 57, 147–159. doi: 10.1016/j.cogsys.2018.12.015
Kumar, T. S., & Vijai, A. (2012). 3d reconstruction of face from 2d ct scan images. Procedia Engineering, 30, 970–977. doi: 10.1016/j.proeng.2012.01.952
Muschelli, J. (2019). Recommendations for processing head ct data. Frontiers in Neuroinformatics, 13, 61. doi: 10.3389/fninf.2019.00061
Schwarz, C. G., Kremers, W. K., Therneau, T. M., Sharp, R. R., Gunter, J. L., Vemuri, P., … Jack, C. R. (2019). Identification of anonymous mri research participants with face-recognition software. New England Journal of Medicine, 381(17), 1684–1686. doi: 10.1056/NEJMc1908881
Wang, S., Liu, Z., Rong, Y., Zhou, B., Bai, Y., Wei, W., … Tian, J. (2019). Deep learning provides a new computed tomography-based prognostic biomarker for recurrence prediction in high-grade serous ovarian cancer. Radiotherapy and Oncology, 132, 171–177. doi: 10.1016/j.radonc.2018.10.019
Wu, T., Sultan, L. R., Tian, J., Cary, T. W., & Sehgal, C. M. (2019). Machine learning for diagnostic ultrasound of triple-negative breast cancer. Breast Cancer Research and Treatment, 173(2), 365–373. doi: 10.1007/s10549-018-4984-7

* Some acronyms lack proper introduction (e.g. EHR).
# EHR definition added (p2), all others checked

*The Authors should also take into consideration another issue: imaging exams may contain identifiable data even in terms of physical characteristics of the patient. For example, a head scan allows easy reconstruction of the subject's face. Making this data public may be problematic if an explicit consent is not given by the patient.

#Precautions against such abuse now detailed on p10. The legal proscription and technical safeguards against any attempt at re-identification of patients are more clearly specified, along with clarification that the image data is never made public, only disclosed to approved researchers within a controlled

research environment with additional safeguards before further disclosure.

* In general, there seems to be no mention of Ethics approval for collecting the data or requesting patient permission to collect data for research purposes. I am not sure of the legal framework in Scotland, but in other European countries informed consent must be given separately for medical procedures and research purposes. Furthermore, consent for research is tied to the aim of the research and should be reissued (or waived by the competent Institutional Ethics Board) on a case-by-case basis. In a table, it is mentioned that researchers who wish to access the data should absolve administrative and ethics requirements. However, there should be an approval (and consent) for collecting the data in the first place.
This is a significant issue and should be appropriately addressed in the text.

Approval issue and additional disclosure controls now explained (p3, p10, p17-18)

* No mention is made of quality control of the input data. Public datasets have shown to have issues both image datasets in general and medical imaging specifically (e.g. doi: 10.1016/j.acra.2019.10.006). Considering these problems arise in curated datasets, how is the risk of bias due to errors (which unfortunately are not rare in clinical practice) addressed? This situation could severely limit the usefulness of the data. Prospectively collected data in the setting of a clinical study has the advantage of greater control of its quality and reduction of bias sources (and is still not perfect).

# Issue acknowledged and tradeoff for size and diversity of data against smaller higher quality research-specific collections added p18. Some mention of quality control issues added, also noting that in some cases concerns such as radiation dosage (CT) make re-use of scans already performed for clinical reasons the only option.

Reviewer 2
* This paper is a useful, readable report upon progress made in the development, design, and delivery of a key component of health informatics infrastructure. The infrastructure in question is intended to facilitate and manage access to routinely-collected image data for research purposes.

* (1) the abstract states that the platform has been tested on five different test cases - it would be good to understand what these test cases are (the brief mention on Page 6 does not convey any real understanding of what was demonstrated)

P5 – some information added, plus explicit reference to Appendix D with full list.

* (2) the way in which the challenges listed at the beginning of the paper are revisited later, to explain how they have been addressed, is very welcome - however, it would be good to have a more detailed explanation in each case

# Summary (p17-18) expanded, with each challenge reiterated and addressed specifically; mention of challenge 1 added.

* (3) the analysis section should be made *more concise and more rigorous*, focussing upon the distinctive aspects of the architecture (rich, extensible metadata, project-specific de-identification)

# Architectural discussion (p7-8) streamlined, less discussion of off-the-shelf elements.

* (4) the existing comments on microservices, message queuing, or even the specific choice of relational database are not particularly informative or useful, and neither are those on test-driven development - it is good to know that engineering principles were applied, but there is not enough information here to inform any evaluation or subsequent application elsewhere - the material could be made more concise, or perhaps simply omitted

# Architectural discussion (p7-8) streamlined.

* (5) in contrast, it would be good to have some detailed information regarding performance and scalability, and to have also some detailed information regarding the two different environments with different hardware and VM tools mentioned on Page 16 - again, there is not enough detail here to add substance to the claim that the software 'proved flexible'

Appendix C rewritten with more platform and performance information.

* (6) the account of the limitations of the architecture is not particularly useful as it stands, and it would be better to address each of these points more clearly as part of the account of the choices made

# Limitations (p16) expanded and refined with mention of mitigation steps and plans.

* (7) the 'discussion' and 'future plans' subsections within 'analysis' could be more concise, and might work better as part of the subsequent 'conclusion' section

Restructured as suggested (p16-18)

* Minor points:
(The page numbers here are those of the reviewer pdf, in which the main document starts with the abstract on Page 3)
Page 4, Line 10: "with a condition" might be better as "with a specific condition"
Page 6, Line 6: "elucidating" might be better as "eliciting"

Minor points both resolved. Also corrected “concurrent” to “consecutive”.

Close
