# Supplementary material for: An extensible big data software architecture managing a research resource of real-world clinical radiology data linked to other health data from the whole Scottish population
Source: Gigascience. 2020 Sep 29;9(10):giaa095. doi: 10.1093/gigascience/giaa095 (PMC7523405; doi:10.1093/gigascience/giaa095)
Supplement: giaa095_Response_to_Reviewer_Comments_Revision_1 [file giaa095_response_to_reviewer_comments_revision_1.pdf]

Author's Response To Reviewer Comments

Close

I have previously provided the point by point response to reviewers and the tracked changes version.
I'm now attaching a version with all tracked changes accepted and the author list and DOIs fixed.

Close
