# Supplementary material for: An extensible big data software architecture managing a research resource of real-world clinical radiology data linked to other health data from the whole Scottish population
Source: Gigascience. 2020 Sep 29;9(10):giaa095. doi: 10.1093/gigascience/giaa095 (PMC7523405; doi:10.1093/gigascience/giaa095)
Supplement: giaa095_Reviewer_1_Report_Original_Submission — Renato Cuocolo -- 4/16/2020 Reviewed [file giaa095_reviewer_1_report_original_submission.pdf]

Reviewer Report

Title: An extensible big data software architecture managing a research resource of real-world clinical radiology data linked to other health data from the whole Scottish population

Version: Original Submission **Date:** 4/16/2020

Reviewer name: Renato Cuocolo

Reviewer Comments to Author:

I thank the Authors for presenting a promising solution for creating a large dataset of medical data, particularly focused on imaging.
Some comments:
The references in the opening of the Background are relatively old (e.g. ref 9 is a review from 2015), there are more recent publications that could be cited (e.g. doi: 10.1186/s41747-019-0109-2).
Some acronyms lack proper introduction (e.g. EHR).
The Authors should also take into consideration another issue: imaging exams may contain identifiable data even in terms of physical characteristics of the patient. For example, a head scan allows easy reconstruction of the subject's face. Making this data public may be problematic if an explicit consent is not given by the patient.
In general, there seems to be no mention of Ethics approval for collecting the data or requesting patient permission to collect data for research purposes. I am not sure of the legal framework in Scotland, but in other European countries informed consent must be given separately for medical procedures and research purposes. Furthermore, consent for research is tied to the aim of the research and should be reissued (or waived by the competent Institutional Ethics Board) on a case-by-case basis. In a table, it is mentioned that researchers who wish to access the data should absolve administrative and ethics requirements. However, there should be an approval (and consent) for collecting the data in the first place.
This is a significant issue and should be appropriately addressed in the text.
No mention is made of quality control of the input data. Public datasets have shown to have issues both image datasets in general and medical imaging specifically (e.g. doi: 10.1016/j.acra.2019.10.006). Considering these problems arise in curated datasets, how is the risk of bias due to errors (which unfortunately are not rare in clinical practice) addressed? This situation could severely limit the usefulness of the data. Prospectively collected data in the setting of a clinical study has the advantage of greater control of its quality and reduction of bias sources (and is still not perfect).

Level of Interest

Please indicate how interesting you found the manuscript: Choose an item.

Quality of Written English

Please indicate the quality of language in the manuscript: Choose an item.

Declaration of Competing Interests

Please complete a declaration of competing interests, considering the following questions:

- Have you in the past five years received reimbursements, fees, funding, or salary from an organisation that may in any way gain or lose financially from the publication of this manuscript, either now or in the future?
- Do you hold any stocks or shares in an organisation that may in any way gain or lose financially from the publication of this manuscript, either now or in the future?
- Do you hold or are you currently applying for any patents relating to the content of the manuscript?
- Have you received reimbursements, fees, funding, or salary from an organization that holds or has applied for patents relating to the content of the manuscript?
- Do you have any other financial competing interests?
- Do you have any non-financial competing interests in relation to this paper?

If you can answer no to all of the above, write 'I declare that I have no competing interests' below. If your reply is yes to any, please give details below.

I declare that I have no competing interests.

I agree to the open peer review policy of the journal. I understand that my name will be included on my report to the authors and, if the manuscript is accepted for publication, my named report including any attachments I upload will be posted on the website along with the authors' responses. I agree for my report to be made available under an Open Access Creative Commons CC-BY license (http://creativecommons.org/licenses/by/4.0/). I understand that any comments which I do not wish to be included in my named report can be included as confidential comments to the editors, which will not be published.

Choose an item.

To further support our reviewers, we have joined with Publons, where you can gain additional credit to further highlight your hard work (see: https://publons.com/journal/530/gigascience). On publication of this paper, your review will be automatically added to Publons, you can then choose whether or not to claim your Publons credit. I understand this statement.

Yes Choose an item.
