# Supplementary material for: An extensible big data software architecture managing a research resource of real-world clinical radiology data linked to other health data from the whole Scottish population
Source: Gigascience. 2020 Sep 29;9(10):giaa095. doi: 10.1093/gigascience/giaa095 (PMC7523405; doi:10.1093/gigascience/giaa095)
Supplement: giaa095_Reviewer_2_Report_Original_Submission — Jim Davies -- 5/20/2020 Reviewed [file giaa095_reviewer_2_report_original_submission.pdf]

Reviewer Report

Title: An extensible big data software architecture managing a research resource of real-world clinical radiology data linked to other health data from the whole Scottish population

Version: Original Submission **Date:** 5/20/2020

Reviewer name: Jim Davies

Reviewer Comments to Author:

This paper is a useful, readable report upon progress made in the development, design, and delivery of a key component of health informatics infrastructure. The infrastructure in question is intended to facilitate and manage access to routinely-collected image data for research purposes.
The paper begins with a brief explanation of the challenges to be addressed and the approach taken to development. This is followed by a description of the solution architecture, as an extension of an existing research data management platform (RDMP) - already reported in this journal - to include support for image data.
The subsequent 'analysis' section comprises:
- a very brief description of the current status (data is being converted into DICOM format, some testing has been performed, and an initial set of tags are supported)
- some justification of technology choices (built upon an existing platform, using a service-based architecture, using a document database and a bespoke relational schema, incorporating an OCR tool)
- some comparison with other platforms (in contrast: metadata-driven, images held in identified form, de-identified versions generated only as required)
- a brief description of the testing strategy (standard software engineering approach, some indications regarding performance and scalability)
- some discussion (an additional remark on the approach to image data management, some assertions regarding re-usability of the software, a general claim regarding potential value)
- short, medium, and long term intentions (plans for more tags, more automation, algorithms for standardisation and classification, shared access to image data)
- some remarks upon limitations of the current architecture (cohort creation still cumbersome, storing images in identified form means more work, relational model may not be ideal)
The final section sets out some brief conclusions: an assertion that the design meets requirements, a report that initial indications suggest that it will work as intended, and a summary of how different aspects of the design are related to the original list of challenges.
The material in the paper is original, and the subject matter is of interest and value. However, some additional work is needed before it is fully ready for publication.
(1) the abstract states that the platform has been tested on five different test cases - it would be good to understand what these test cases are (the brief mention on Page 6 does not convey any real understanding of what was demonstrated)
(2) the way in which the challenges listed at the beginning of the paper are revisited later, to explain how they have been addressed, is very welcome - however, it would be good to have a more detailed

explanation in each case
(3) the analysis section should be made *more concise and more rigorous*, focussing upon the distinctive aspects of the architecture (rich, extensible metadata, project-specific de-identification)
(4) the existing comments on microservices, message queuing, or even the specific choice of relational database are not particularly informative or useful, and neither are those on test-driven development - it is good to know that engineering principles were applied, but there is not enough information here to inform any evaluation or subsequent application elsewhere - the material could be made more concise, or perhaps simply omitted
(5) in contrast, it would be good to have some detailed information regarding performance and scalability, and to have also some detailed information regarding the two different environments with different hardware and VM tools mentioned on Page 16 - again, there is not enough detail here to add substance to the claim that the software 'proved flexible'
(6) the account of the limitations of the architecture is not particularly useful as it stands, and it would be better to address each of these points more clearly as part of the account of the choices made
(7) the 'discussion' and 'future plans' subsections within 'analysis' could be more concise, and might work better as part of the subsequent 'conclusion' section
Minor points:
(The page numbers here are those of the reviewer pdf, in which the main document starts with the abstract on Page 3)
Page 4, Line 10: "with a condition" might be better as "with a specific condition"
Page 6, Line 6: "elucidating" might be better as "eliciting"

Level of Interest

Please indicate how interesting you found the manuscript: Choose an item.

Quality of Written English

Please indicate the quality of language in the manuscript: Choose an item.

Declaration of Competing Interests

Please complete a declaration of competing interests, considering the following questions:

- Have you in the past five years received reimbursements, fees, funding, or salary from an organisation that may in any way gain or lose financially from the publication of this manuscript, either now or in the future?
- Do you hold any stocks or shares in an organisation that may in any way gain or lose financially from the publication of this manuscript, either now or in the future?
- Do you hold or are you currently applying for any patents relating to the content of the manuscript?

- Have you received reimbursements, fees, funding, or salary from an organization that holds or has applied for patents relating to the content of the manuscript?
- Do you have any other financial competing interests?
- Do you have any non-financial competing interests in relation to this paper?

If you can answer no to all of the above, write 'I declare that I have no competing interests' below. If your reply is yes to any, please give details below.

I declare that I have no competing interests.

I agree to the open peer review policy of the journal. I understand that my name will be included on my report to the authors and, if the manuscript is accepted for publication, my named report including any attachments I upload will be posted on the website along with the authors' responses. I agree for my report to be made available under an Open Access Creative Commons CC-BY license (http://creativecommons.org/licenses/by/4.0/). I understand that any comments which I do not wish to be included in my named report can be included as confidential comments to the editors, which will not be published.

Choose an item.

To further support our reviewers, we have joined with Publons, where you can gain additional credit to further highlight your hard work (see: https://publons.com/journal/530/gigascience). On publication of this paper, your review will be automatically added to Publons, you can then choose whether or not to claim your Publons credit. I understand this statement.

Yes Choose an item.
